# The combinatorial action of hyphal growth and candidalysin is critical for promoting *Candida albicans* oropharyngeal infection

**DOI:** 10.1128/mbio.03304-25

**Published:** 2025-11-26

**Authors:** Olivia K. A. Paulin, Antzela Tsavou, Emily L. Priest, James S. Griffiths, Léa Lortal, Alexander Kempf, Eve W. L. Chow, Li Mei Pang, Don N. Wickramasinghe, Claire M. Lyon, Aaron D. Hernday, Yue Wang, Jonathan P. Richardson, Julian R. Naglik

**Affiliations:** 1 Centre for Host-Microbiome Interactions, Faculty of Dentistry, Oral & Craniofacial Sciences, King's College London 61139 , London, United Kingdom; 2 A*STAR Infectious Diseases Laboratories (A*STAR IDL), Agency for Science Technology and Research (A*STAR) 54759, Singapore, Singapore; 3 Department of Molecular and Cell Biology, University of California Merced 33244, Merced, California, USA; 4 Health Sciences Research Institute, University of California 33244, Merced, California, USA; Instituto Carlos Chagas, Curitiba, Brazil

**Keywords:** *Candida albicans*, infectious disease, CRISPR, host-pathogen interactions, oropharyngeal candidiasis

## Abstract

*Candida albicans* is one of the most common fungal pathogens, yet much remains unknown about how its virulence factors cooperate to promote pathogenicity. To investigate this, CRISPR-Cas9 technology was used to create a panel of 19 single, double, triple, and quadruple deletion mutant strains targeting four established virulence factors: *ALS3* (adhesin/invasin), *ECE1* (candidalysin toxin), *HGC1* (hypha formation regulator), and *SAP2* (protease). *In vitro*, the deletion of each gene had differing impacts across multiple characterization assays. The *hgc1*∆/∆ mutant was unable to form hyphae under inducing conditions, leading to downstream impairment of epithelial invasion. The *als3*∆/∆ mutant exhibited significantly reduced adhesion and invasion into epithelial cells, resulting in attenuated cellular damage. The *ece1*∆/∆ mutant displayed significantly reduced epithelial damage, cell signaling, and immune activation. The phenotype of the *sap2*∆/∆ mutant resembled that of wild type but was unable to degrade protein. In an immunocompromised murine model of oropharyngeal infection, hyphal growth and candidalysin production were the dominant drivers of elevated fungal burden, innate immune responses, and mortality. Following a 5-day infection with *hgc1*∆/∆ and *ece1*∆/∆ single gene deletion strains, mice had survival rates of 100% and 80%, respectively, compared to 15% in wild-type infected mice. Notably, 100% survival was also observed following challenge with all *hgc1*∆/∆ and *ece1*∆/∆ combination mutants. This study demonstrates that specific *C. albicans* virulence attributes act in combination to promote mucosal infection, with hyphal growth and candidalysin production being a critical driver of oropharyngeal infection.

**IMPORTANCE:**

*Candida albicans* has been classified by the WHO as a “critical priority” pathogen, highlighting the urgent need for a greater understanding of the mechanisms that enable it to cause disease. *C. albicans* possesses numerous virulence attributes, but how they synergize during infection is not well understood. Here, using reverse genetics, we dissect the individual and combinatorial roles of four *C. albicans* virulence factors (Als3p, candidalysin, hyphal growth, and Sap2p) *in vitro* and in an *in vivo* murine model of oropharyngeal candidiasis. Increasing the number of *C. albicans* gene deletions correlated with reduced oral fungal burden, with hyphal growth and candidalysin together being critical for infection, inflammation, and mortality during oropharyngeal infection. These findings demonstrate that virulence attributes act cooperatively as a collective network to promote pathogenicity, a finding also observed in plant fungal pathogens. Our approach has identified specific fungal virulence factors that can be targeted for new treatment strategies against *C. albicans* infections.

## INTRODUCTION

*Candida albicans* is a commensal fungus frequently found in the microbiota of the oral, vaginal, and gut mucosa (1). Under predisposing conditions, overgrowth and localized tissue invasion promote infection (2). This is of particular concern in the immunocompromised population, in which dissemination to the systemic compartment carries an unacceptably high mortality rate of approximately 40% (3).

*C. albicans* infection is driven by its virulence factors. First, *C. albicans* can adapt to varied host environments by switching morphologies from yeast to hypha (2). Host factors such as elevated temperature, pH fluctuations, and CO_2_ concentrations all trigger hyphal switching, facilitated by many genes and transcriptional regulators (2). Specifically, the *HGC1* gene encodes a G1 cyclin-related protein that is integral for morphological switching and hyphal maintenance (4). Critically, a *hgc1*∆/∆ mutant is unable to form hyphae under hypha-inducing conditions, but can still express hypha-associated genes, including *ALS3*, *HWP1,* and *ECE1* (2, 4).

Once in the hyphal state, *C. albicans* adheres strongly to mucosal tissue and invades via two distinct, yet complementary mechanisms: active penetration and induced endocytosis (5). The *ALS* gene family is integral for induced endocytosis (6). Within this family, Als3p plays the most direct role in hyphal adhesion by binding to both E-cadherin and the EGFR/Her2 heterodimeric receptor complex (6). The formation of Als3p/E-cadherin and Als3p/EGFR/Her2 complexes stimulates cytoskeletal remodeling within the host epithelial cell, facilitating hyphal invasion and creating an “invasion pocket” into which candidalysin is secreted (7). Candidalysin is a peptide toxin that exists as a discrete sequence within a larger preproprotein encoded by the gene *ECE1* (8). Proteolytic processing of Ece1p produces a mature candidalysin, which is secreted from *C. albicans* hyphae (8). Candidalysin plays a key role in virulence by damaging epithelial cells and stimulating mitogen-activated protein kinase (MAPK) signaling and subsequent innate immune responses (8, 9).

Other virulence factors *C. albicans* utilizes include its hydrolytic enzymes such as lipases, phospholipases, and secreted aspartyl proteases (Saps) (10). Of these, Sap2p is the most abundantly secreted during oral infection in humans (11, 12) and degrades numerous host molecules, including IgA and mucin, thereby reducing host barrier function and facilitating fungal invasion (10, 13).

To assess the role of specific genes in microbial virulence, knockout studies are often performed. This “reverse genetics” approach provides crucial information about gene function, particularly in the context of pathogenicity. CRISPR has streamlined mutant construction in diploid *C. albicans* strains, as it can create homozygous gene knockouts in a single transformation (14). The CRISPR system used for this work has the advantage of marker recycling, facilitating sequential rounds of transformation in the same mutated strain following the excision of the integration cassette (15).

While single knockout studies of genes encoding key virulence factors are common, our understanding of the combinatorial action of virulence factors in an infection setting remains limited. Additionally, accurate phenotypic comparisons between gene knockout studies are often hindered by different genetic backgrounds (16, 17). To mitigate these issues, we used CRISPR-Cas9 to construct a panel of single and combinatorial homozygous gene knockouts in *C. albicans ALS3*, *ECE1*, *HGC1,* and *SAP2* in a single genetic background and assessed the ability of these mutant strains to cause disease.

Consistent with previous studies, *ALS3* deletion reduced adhesion, invasion, and host damage; *ECE1* deletion attenuated damage, MAPK signaling, and immune activation; and *HGC1* deletion prevented hyphal growth, impairing invasion. The deletion of *SAP2* prevented protein degradation but had no impact on host responses *in vitro*. These trends were sustained when gene targets were deleted in combination, with additive effects also being observed. In an immunocompromised murine model of oropharyngeal candidiasis (OPC), the most important virulence attributes were the combined action of hypha formation and candidalysin secretion. Indeed, OPC induced with any mutant strain that included a dual deletion of *HGC1* and *ECE1* exhibited significantly reduced fungal burden, immune responses, and mortality. Notably, while deletion of *ALS3* and *SAP2* alone had minimal impact on survival, the deletion of both genes strongly impaired virulence, resulting in a significant increase in survival.

These data delineate the virulence attributes that are most important for driving OPC, revealing that they act in combination to promote infection. Specifically, our findings identify hypha formation and candidalysin as key virulence attributes that could be targeted for new treatment strategies against mucosal *C. albicans* infections.

## RESULTS

### Construction of mutant strains

**Fig 1:**
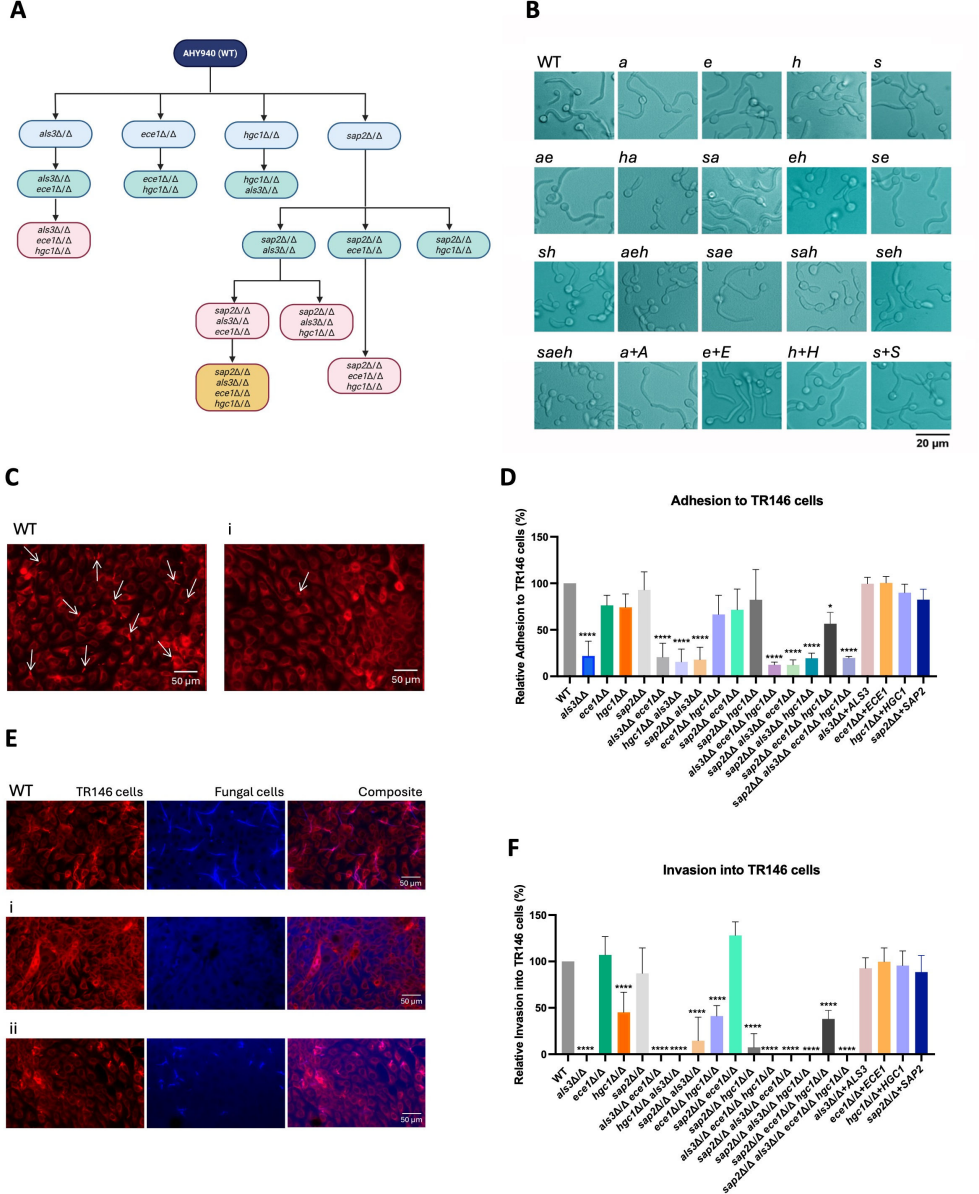
Hgc1p is required for hyphal morphogenesis and epithelial invasion; Als3p is crucial for epithelial adhesion and invasion. (**A**) Order of mutation for each CRISPR deletion mutant constructed for this study. The background strain of AHY940 (WT) was used for all strains, with additive deletions being performed on mutated strains following CRISPR cassette recycling. Created with BioRender. (**B**) Strains were grown in yeast peptone dextrose + 10% fetal bovine serum at 37°C and 200 RPM for 3 h to induce hyphal growth. Strains presented: AHY940 (WT), *als3*Δ/Δ (a)*, ece1*Δ/Δ (e)**,** *hgc1*Δ/Δ (h)**,** *sap2*Δ/Δ (s)**,** *als3*Δ/Δ *ece1*Δ/Δ (*ae*)**,** *hgc1*Δ/Δ *als3*Δ/Δ (*ha*)**,** *sap2*Δ/Δ *als3*Δ/Δ (*sa*)**,** *ece1*Δ/Δ *hgc1*Δ/Δ (*eh*)*, sap2*Δ/Δ *ece1*Δ/Δ (*se*)**,** *sap2*Δ/Δ *hgc1*Δ/Δ (*sh*), *als3*Δ/Δ *ece1*Δ/Δ *hgc1*Δ/Δ (*aeh*), *sap2*Δ/Δ *als3*Δ/Δ *ece1*Δ/Δ (*sae*), *sap2*Δ/Δ *als3*Δ/Δ *hgc1*Δ/Δ (*sah*), *sap2*Δ/Δ *ece1*Δ/Δ *hgc1*Δ/Δ (*seh*), *sap2*Δ/Δ *als3*Δ/Δ *ece1*Δ/Δ *hgc1*Δ/Δ (*saeh*), *als3*Δ/Δ + *ALS3* (*a*+*A*), *ece1*Δ/Δ + *ECE1* (*e*+*E*), *hgc1*Δ/Δ + *HGC1* (*h*+*H*), and *sap2*Δ/Δ + *SAP2* (*s*+*S*). TR146 epithelial cells were infected with strains (multiplicity of infection 1) for (**C and D**) 1 h or (**E and F**) 4 h before staining with concanavalin-A and calcofluor white. (**C and D**) The number of fungal cells adhered was enumerated, and data are presented as (**C**) representative microscopy images of AHY940 (WT) and *als3*Δ/Δ (i) (with arrows pointing at selected fungal cells) and (**D**) percent adhesion relative to WT. (**E**) Representative invasion microscopy images of AHY940 (WT), *als3*Δ/Δ (i), and *hgc1*Δ/Δ (ii) presented as TR146 cells, fungal cells, and a composite image. (**F**) Percent invasion relative to WT. Statistical significance was calculated using a one-way ANOVA using a Dunnett’s comparison test fixed to AHY940 (WT). ****P > 0.0001; *P > 0.05. Scale bar represents (**B**) 20 µm or (**C and E**) 50 µm. All data are representative of three biological repeats.

To assess the role of *ALS3*, *ECE1*, *HGC1,* and *SAP2* in *C. albicans* virulence, a panel of 19 mutant strains comprising homozygous single, double, triple, and quadruple gene deletions was created in a single genetic background using CRISPR-Cas9 genetic editing, with one sgRNA per gene (Fig. 1A). The entire open reading frame (ORF) of *ALS3*, *ECE1,* and *HGC1* was deleted, and the lack of gene expression was confirmed by reverse transcription quantitative real-time PCR (RT-qPCR) (Fig. S1A through C). Despite several attempts and approaches, a whole ORF deletion of *SAP2* proved unsuccessful. To mitigate this issue, a premature in-frame stop codon was introduced at amino acid position 59 of the *SAP2* ORF to abolish Sap2p production. Accordingly, the *sap2*∆/∆ mutants were characterized using a protein digestion assay (18) to confirm the absence of Sap2p activity (Fig. S1Di).

Following mutant construction, we sought to investigate whether the deletion of *ALS3*, *ECE1*, *HGC1,* or *SAP2* induced compensatory expression changes of the other three selected genes (i.e., *ECE1*, *HGC1,* and *SAP2* in *als3*∆/∆). Mutant strains were cultured under yeast and hypha-inducing conditions, and gene expression was quantified by RT-qPCR (Fig. S1A through C and E). As expected, *ALS3* and *ECE1* gene expression was reduced (but only minimally) in the mutants harboring a *HGC1* deletion, as *HGC1* regulates hyphal formation/maintenance (4). *HGC1* and *SAP2* gene expression was unaffected in all other mutants not containing a *HGC1* or *SAP2* deletion, respectively. For Sap2p activity, the quadruple mutant was additionally tested to confirm the lack of Sap2p activity, indicating the premature in-frame stop codon was retained in all mutants (Fig. S1D).

The *ALS* and *SAP* gene families contain 8 and 10 associated genes, respectively, which have partial functional redundancy and differing essentiality for virulence (13, 19–21). It has been reported that genes within a family can compensate for specific gene knockouts by altering expression levels (22). To investigate if compensatory changes in *ALS* and *SAP* expression were occurring in *als3*Δ/Δ and *sap2*Δ/Δ mutant strains, RT-qPCR was performed. For *ALS* gene expression, five representative strains were selected for analysis, including wild-type (WT), a*ls3*Δ/Δ, *hgc1*Δ/Δ a*ls3*Δ/Δ, *sap2*Δ/Δ a*ls3*Δ/Δ *ece1*Δ/Δ *hgc1*Δ/Δ and a*ls3*Δ/Δ + *ALS3*. These data revealed no significant differences between *ALS1*, *ALS4-7,* and *ALS9* gene expression in any of the mutant strains cultured under yeast and hypha-inducing conditions (Fig. S2A, C through G). Only *ALS2* gene expression appeared to show variations between strains (Fig. S2B). Indeed, in the hypha-grown conditions, a*ls3*Δ/Δ and *sap2*Δ/Δ a*ls3*Δ/Δ *ece1*Δ/Δ *hgc1*Δ/Δ had modest but significantly elevated *ALS2* expression levels compared to WT (1.8- and 2.1-fold increase, respectively) (Fig. S2B). In the yeast-grown conditions, *hgc1*Δ/Δ a*ls3*Δ/Δ had significantly elevated *ALS2* expression levels compared to a*ls3*Δ/Δ, *sap2*Δ/Δ a*ls3*Δ/Δ *ece1*Δ/Δ *hgc1*Δ/Δ, and a*ls3*Δ/Δ + *ALS3* (2.2-fold increase; 1.7-fold increase; and 2.2-fold increase, respectively) (Fig. S2B).

The expression of *SAP1* and *SAP3-10* was quantified in the following strains: WT, *sap2*Δ/Δ, *sap2*Δ/Δ *hgc1*Δ/Δ, *sap2*Δ/Δ a*ls3*Δ/Δ *ece1*Δ/Δ *hgc1*Δ/Δ, and *sap2*Δ/Δ + *SAP2*. Analysis revealed that across all genes, there were no significant differences in expression between any of the strains in yeast or hyphal growth conditions (Fig. S3).

### *HGC1* is essential for hyphal morphogenesis, while *ALS3* is crucial for adhesion and invasion

To characterize the panel of mutant strains, morphology was assessed under liquid hypha-inducing conditions, as this is a key hallmark of *C. albicans* virulence. Mutant strains were cultured in yeast peptone dextrose (YPD) medium + 10% fetal bovine serum (FBS) at 37°C with shaking and were analyzed by microscopy after 3 h (2). This revealed that the *hgc1*Δ/Δ strains were unable to form elongated hyphae, as only short germ tubes were visible. All other strains formed elongated hyphae, including the *HGC1* re-integrant strain (Fig. 1B). These data confirm that Hgc1p plays a key role in regulating the yeast-to-hyphal morphological switch in *C. albicans* (4).

**Fig 2:**
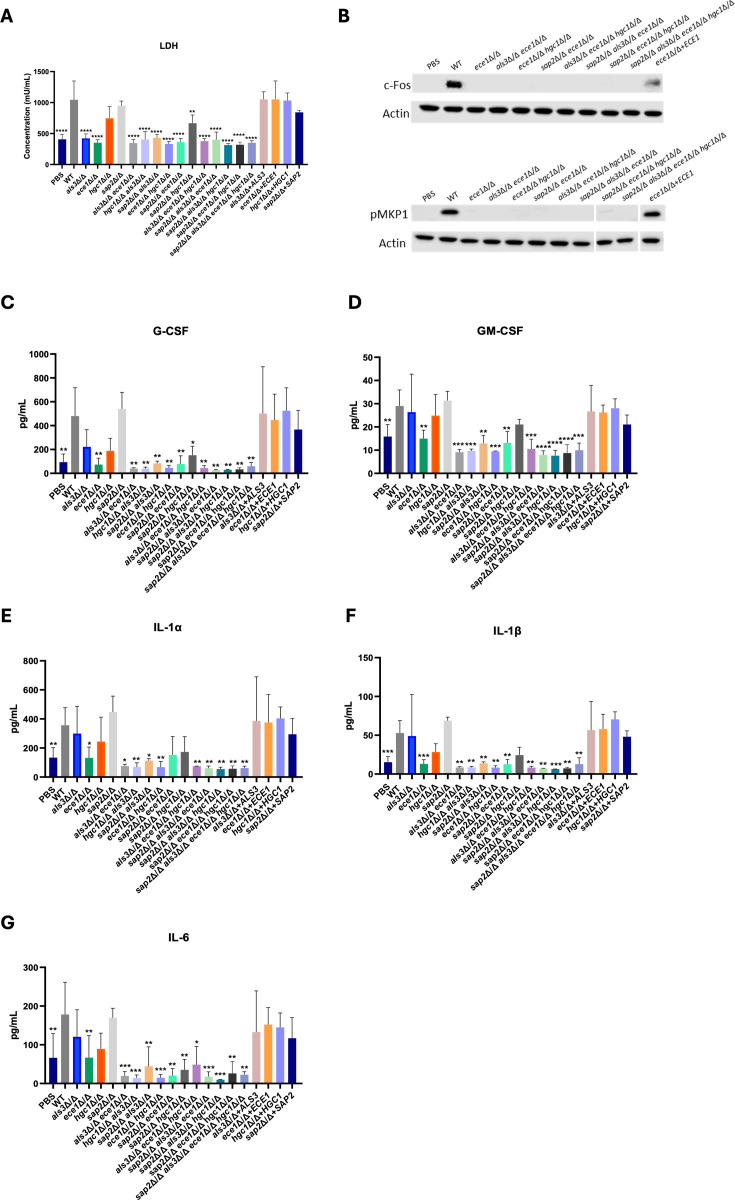
*C. albicans ece1*∆**/**∆ mutant strains have severely attenuated ability to cause epithelial damage, MAPK signaling, and immune activation. (**A**) TR146 cells were infected with Dulbecco’s phosphate-buffered saline (PBS), AHY940 (WT), and all mutant strains for 24 h (multiplicity of infection [MOI] 0.01), and cellular damage was quantified via lactate dehydrogenase (LDH) activity. (**B**) TR146 cells were infected with PBS, AHY940 (WT), and all *ece1*∆/∆ mutant strains for 2 h (MOI 10). Samples containing 10 µg of total protein were loaded onto SDS-PAGE 4%–12% gradient gels to detect c-Fos expression and MKP1 phosphorylation. α-actin was probed as a loading control. White dividing lines represent a cut on the same blot to rearrange sample order. (**C**) TR146 epithelial cells were infected with PBS, WT (AHY940), and all mutant strains for 24 h, and secretion of granulocyte colony-stimulating factor (G-CSF), (**D**) granulocyte-macrophage colony-stimulating factor (GM-CSF), (**E**) IL-1α, (**F**) IL-1β, and (**G**) IL-6 was quantified. Data are the mean and standard deviation from three biological replicates. Statistical significance was calculated using one-way ANOVA and a Dunnett’s comparison test compared to WT. ****P > 0.0001; ***P > 0.001; **P > 0.01; and *P > 0.05.

As Als3p binds to E-cadherin and the EGFR/Her2 complex on epithelial cells (23, 24), all mutant strains were assessed for their ability to adhere to TR146 cells. Epithelial cells were infected with mutant strains for 1 h, then fixed and stained for microscopy analysis (Fig. 1C and D). Compared to WT, the adherence of the *als3*Δ/Δ mutant was significantly reduced by 78.1% (Fig. 1D). This was similarly observed in all other *als3*Δ/Δ multi-deletion strains. This defect was restored to WT levels in the *als3*Δ/Δ + *ALS3* re-integrant strain. All other strains had adhesion comparable to WT, except for the *sap2*Δ/Δ *ece1*Δ/Δ *hgc1*Δ/Δ triple mutant, which had significantly less adhesion than WT (43% reduction) (Fig. 1D). These data confirm that Als3p is the key mediator of adhesion to epithelial cells, but a triple deletion of *sap2*Δ/Δ *ece1*Δ/Δ *hgc1*Δ/Δ also negatively impacted adherence. Microscopy images for all other mutant strains are presented in Fig. S4.

Next, invasion into TR146 epithelial cells was assessed, as Als3p induces endocytosis of the fungal cell (6). Strains were incubated with TR146 cells for 4 h, then fixed and stained with concanavalin-A and calcofluor white. Microscopy analysis revealed that WT *C. albicans* had an invasion rate of 51% into the epithelial cells (Fig. 1E and F). In contrast, no fungal cells were observed in any of the *als3*∆/∆ strain images, ascribed to them being largely non-adherent and therefore removed during the staining process, directly impacting invasion (Fig. 1Ei and F). The *hgc1*∆/∆ single deletion mutant had a significantly lower invasion rate of 21% (Fig. 1Eii and F). This defect was also observed in the multi-deletion mutants containing the *hgc1*∆/∆ mutation, such as *ece1*∆/∆ *hgc1*∆/∆, *sap2*∆/∆ *hgc1*∆/∆, and *sap2*∆/∆ *ece1*∆/∆ *hgc1*∆/∆ (19%, 4%, and 17%, respectively) (Fig. 1F and Fig. S5). All other knockout and re-integrated strains had invasion rates similar to WT (Fig. 1F and Fig. S5). Collectively, these data confirm that Als3p and Hgc1p are critical for *C. albicans* invasion into epithelial cells, which is conserved across all multi-deletion mutants lacking *ALS3* and/or *HGC1*.

### All *ece1***∆/∆** mutant strains cause attenuated cellular damage, signaling, and immune activation

A key characteristic of *C. albicans* infection is its dependence on candidalysin to damage the epithelium, activate signaling pathways, and induce innate immune responses (8). To determine whether the mutant strains were able to elicit damage, TR146 cells were infected with all mutant strains for 24 h (multiplicity of infection [MOI] 0.01), and lactate dehydrogenase (LDH) activity (a surrogate marker of cellular damage) was measured in exhausted culture medium. As expected, significant levels of damage were observed following infection with WT in contrast to all the *ece1*Δ/Δ-containing mutant strains, which did not induce damage (comparable to vehicle-treated cells). The *ece1*Δ/Δ + *ECE1* re-integrant strain caused cellular damage comparable to WT (Fig. 2A). These observations confirmed that epithelial cell damage is *ECE1*-dependent. It should be noted that all *ALS3-*deletion strains also caused significantly lower levels of damage; however, we suggest that this was due to a lack of adherence and invasion into the epithelial cells, thereby reducing levels of candidalysin secretion into the invasion pocket (Fig. 1D and F). Interestingly, despite the *hgc1*Δ/Δ and *sap2*Δ/Δ single mutants not differing from WT in their ability to cause damage, the *sap2*Δ/Δ *hgc1*Δ/Δ double mutant caused significantly less damage than WT, indicating a cumulative loss of damage with additive deletion.

The epithelial MAPK signaling pathway is activated by candidalysin, leading to downstream expression of c-Fos and the phosphorylation of MKP1 (25). Therefore, c-Fos expression and MKP1 phosphorylation were assessed to determine whether *ece1*Δ/Δ mutant strains could induce MAPK signaling. TR146 cells were infected with strains for 2 h (MOI 10), after which cell lysate was collected and analyzed by western blot. Infection with WT *C. albicans* strongly induced c-Fos expression and MKP1 phosphorylation. In contrast, epithelial responses to all *ece1*Δ/Δ mutant strains resembled the vehicle-infected control group (Fig. 2B). Densitometry analysis confirmed that c-Fos was not expressed and MKP1 was not phosphorylated in response to infection with *ece1*Δ/Δ-containing mutant strains, but both were restored to WT-like levels in response to the *ECE1* re-integrant strain (Fig. S6A and B). These data confirm that *C. albicans ECE1* is required to induce epithelial MAPK signaling.

Activation of epithelial cells and innate immune signaling at mucosal surfaces is a critical aspect of the host response to *C. albicans* infection and is driven by candidalysin (26). Signaling pathways, including ERK1/2- and p38-MAPK, culminate in the release of pro-inflammatory cytokines (25). To investigate epithelial cytokine responses, TR146 cells were infected for 24 h with mutant and re-integrant strains (MOI 0.01), and secretion of granulocyte colony-stimulating factor (G-CSF), granulocyte-macrophage colony-stimulating factor (GM-CSF), interleukin (IL)-1α, IL-1β, and IL-6 was quantified (Fig. 2C through G).

We observed high levels of G-CSF secretion from WT-infected epithelial cells. In contrast, epithelial cells infected with the *ece1*∆/∆ mutant resembled the vehicle-treated control (Fig. 2C). Interestingly, although none of the other single deletion mutants (*hgc1*Δ/Δ, *als3*Δ/Δ, *sap2*Δ/Δ) caused a significant reduction in G-CSF levels, infection with all the multi-deletion mutants led to significantly reduced secretion compared to WT. Across all re-integrant strains, G-CSF levels were comparable to WT-infected cells.

Next, GM-CSF, IL-1α, and IL-1β were investigated (Fig. 2D through F). Epithelial cells infected with vehicle or *ece1*Δ/Δ displayed significant reductions in secretion compared to WT, unlike any other single deletion mutants. Infection with all the multi-deletion mutants except *sap2*∆/∆ *hgc1*∆/∆ (GM-CSF, IL-1α, and IL-1β) and *sap2*∆/∆ *ece1*∆/∆ (IL-1α) led to a significant reduction in cytokine secretion.

Finally, epithelial cells infected with *ece1*∆/∆ secreted significantly less IL-6 in comparison to WT-treated cells, resembling the vehicle-treated control. Infection with the multi-deletion mutants also caused significant reductions in IL-6 secretion compared to WT, despite infection with *als3*∆/∆, *hgc1*∆/∆, and *sap2*∆/∆ single mutants not causing significant differences (Fig. 2G).

Overall, these data sets indicate that Ece1p plays the most prominent role in eliciting epithelial damage, MAPK signaling, and immune activation; however, Als3p, Hgc1p, and Sap2p may also play a cumulative role in these processes.

### Hyphal growth and candidalysin are required for fungal infection in a murine model of OPC

We next assessed the virulence of the mutant strains in a murine model of OPC, measuring fungal burden, immune activation, and survival. Due to the severity of combined immunosuppression and OPC infection, mice were harvested once experimental limits were reached, up to day 5. Colony-forming units (CFU) were determined on the day of death (with time points clearly stated), indicating that different mutant strains had varying abilities to cause mucosal infection. However, since mice succumbed to infection on different days, the experiment was repeated with a fixed endpoint on day 2 for all mice.

**Fig 3:**
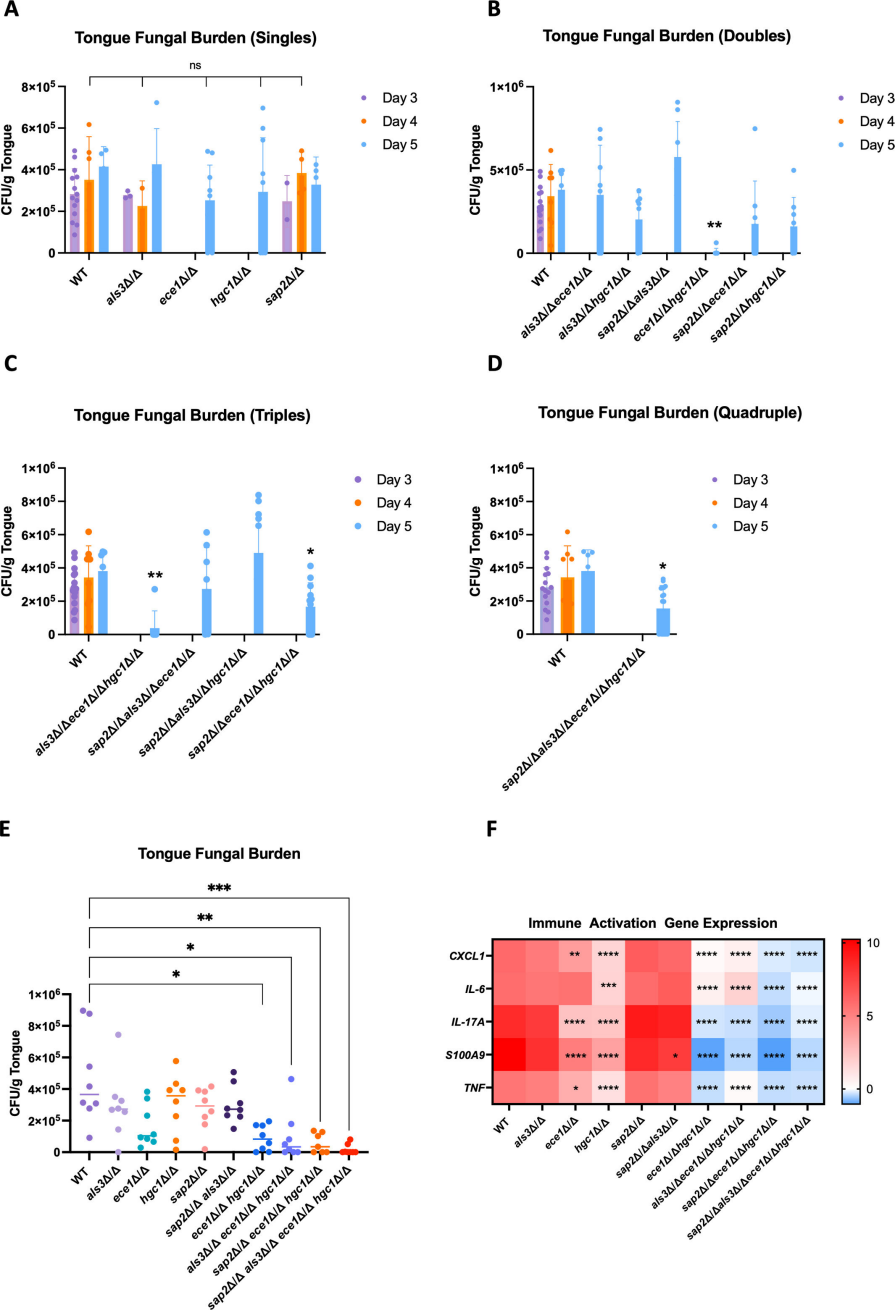
Hyphal growth and candidalysin are required for elevated tongue fungal burden and immune activation. Fungal colony-forming units and gene expression levels in the tongue were assessed following a (**A–D**) 5-day or (**E and F**) 2-day OPC infection in immunocompromised mice. Mice were infected sublingually for 75 min with cotton swabs soaked in a solution of 1 × 10^7^ cells/mL of different *C. albicans* mutant strains, and tongues were harvested on day 2 (**E and F**), day of death (**A–D**), or day 5 post-infection (**A–D**). Data are separated into infection with (**A**) single, (**B**) double, (**C**) triple, and (**D**) quadruple deletion mutants compared to WT (AHY940) or day-2 (**E**) fungal burden and (**F**) host gene expression levels following infection with AHY940 (WT), *als3*Δ/Δ, *ece1*Δ/Δ, *hgc1*Δ/Δ, *sap2*Δ/Δ, *sap2*Δ/Δ *als3*Δ/Δ, *ece1*Δ/Δ *hgc1*Δ/Δ, *als3*Δ/Δ *ece1*Δ/Δ *hgc1*Δ/Δ, *sap2*Δ/Δ *ece1*Δ/Δ *hgc1*Δ/Δ, and *sap2*Δ/Δ *als3*Δ/Δ *ece1*Δ/Δ *hgc1*Δ/Δ. (**A–E**) Data are presented as individual values obtained per mouse (*n* = 8–10) across two biological repeats, with median values depicted as (**A–D**) the top of the bar or (**E**) a horizontal line. (**F**) Heatmap depicting RT-qPCR gene expression data sets for genes encoding immune components. Data are presented as log_2_ fold change *CXCL1*, *IL-6*, *IL-17A*, *S100A9,* and *TNF* gene expression relative to a naive control. (**A–D**) Statistical significance was calculated using the mean of all data points over the 3 days (where applicable) to assess the main row effect, and a two-way ANOVA with Dunnett’s comparison test fixed to AHY940 (WT) was applied. (**E**) Statistical significance was calculated using a non-parametric Kruskal-Wallis test with Dunnett’s comparison test fixed to AHY940 (WT). (**F**) Statistical significance was calculated by comparing the mean of each row using a two-way ANOVA with a Sidak’s multiple comparison test. ****P < 0.0001; ***P < 0.001; **P < 0.01; and *P < 0.05.

First, the single mutants were assessed alongside WT (AHY940), and tongues of infected mice were harvested on the day of death or day 5 for oral fungal burden analysis, revealing no differences in CFU between WT and any of the single deletion mutants (Fig. 3A). Next, all double mutants were assessed for CFU from tongues enumerated on day 5 (Fig. 3B). Infection of the tongue tissue was not observed following challenge with the *ece1*Δ/Δ *hgc1*Δ/Δ mutant. In contrast, challenge with *als3*Δ/Δ *ece1*Δ/Δ suggested a mild increase in fungal burden compared to WT (WT: 3.08 × 10^5^ CFU/g; *als3*Δ/Δ *ece1*Δ/Δ: 3.90 × 10^5^ CFU/g), whereas challenge with *hgc1*Δ/Δ *als3*Δ/Δ suggested a mild reduction in CFU (2.83 × 10^5^ CFU/g), albeit not significant compared to WT-treated mice. The highest fungal burden observed across all double mutants was with *sap2*Δ/Δ *als3*Δ/Δ, with a median value of 5.04 × 10^5^ CFU/g tissue, although this was not statistically significant compared to WT-infected mice (WT: 3.08 × 10^5^ CFU/g). Mice infected with *sap2*Δ/Δ *ece1*Δ/Δ had a fungal burden of 8.34 × 10^4^ CFU/g, 3.7-fold lower than WT, whereas *sap2*Δ/Δ *hgc1*Δ/Δ-challenged mice had a 2.3-fold decrease in fungal burden compared to WT (1.36 × 10^5^ CFU/g) (Fig. 3B).

Next, we assessed the triple mutants for their ability to cause infection (day 5 data only) (Fig. 3C). Infection of tongue tissue was not observed following challenge with the *als3*Δ/Δ *ece1*Δ/Δ *hgc1*Δ/Δ mutant (median value of 0 CFU/g). In contrast, mice challenged with *sap2*Δ/Δ *als3*Δ/Δ *ece1*Δ/Δ had a fungal burden similar to the WT group (WT: 3.08 × 10^5^ CFU/g; *sap2*Δ/Δ *als3*Δ/Δ *ece1*Δ/Δ: 2.93 × 10^5^ CFU/g). Mice challenged with *sap2*Δ/Δ *als3*Δ/Δ *hgc1*Δ/Δ had elevated fungal burdens compared to WT-challenged mice with a CFU of 3.96 × 10^5^ CFU/g, resembling the *als3*Δ/Δ *ece1*Δ/Δ-infected mice. In contrast, *sap2*Δ/Δ *ece1*Δ/Δ *hgc1*Δ/Δ-challenged mice had a significantly lower fungal burden than WT-infected mice (WT: 3.08 × 10^5^ CFU/g; *sap2*Δ/Δ *ece1*Δ/Δ *hgc1*Δ/Δ: 1.98 × 10^5^ CFU/g) (Fig. 3C).

Finally, we investigated the *sap2*Δ/Δ *als3*Δ/Δ *ece1*Δ/Δ *hgc1*Δ/Δ mutant, which, similarly to *sap2*Δ/Δ *ece1*Δ/Δ *hgc1*Δ/Δ, caused a fungal burden of 1.98 × 10^5^ CFU/g, a significant reduction compared to the WT group (WT: 3.08 × 10^5^ CFU/g) (Fig. 3D).

Overall, these data indicate that the combination of hyphal growth and candidalysin production (demonstrated by the deletion of *HGC1* and *ECE1*) is vital for driving infection in the tongue, with only a minimal contribution provided by *ALS3* and *SAP2*.

Next, fungal burden was assessed on day 2 in strains that displayed clear CFU differences compared to WT in the day 3–5 data sets. The selected strains included *als3*Δ/Δ, *ece1*Δ/Δ, *hgc1*Δ/Δ, *sap2*Δ/Δ, *sap2*Δ/Δ *als3*Δ/Δ, *ece1*Δ/Δ *hgc1*Δ/Δ, *als3*Δ/Δ *ece1*Δ/Δ *hgc1*Δ/Δ, *sap2*Δ/Δ *ece1*Δ/Δ *hgc1*Δ/Δ, and *sap2*Δ/Δ *als3*Δ/Δ *ece1*Δ/Δ *hgc1*Δ/Δ (henceforth termed “the selected panel” throughout this study).

On day 2 post-infection, the observed CFU in mice challenged with WT was 3.65 × 10^5^/g. Infection with *als3*Δ/Δ, *hgc1*Δ/Δ, and *sap2*Δ/Δ resembled WT-treated mice with median CFUs of 2.73 × 10^5^/g, 3.57 × 10^5^/g, and 2.93 × 10^5^/g, respectively. In contrast, infection with *ece1*Δ/Δ led to a reduction in CFU (1.03 × 10^5^/g), albeit this was non-significant (Fig. 3E).

In the day 3–5 data set, challenge with *sap2*Δ/Δ *als3*Δ/Δ resulted in the highest observed CFU across all mutant strains (Fig. 3B); however, this elevation was not observed on day 2 (WT: 3.65 × 10^5^ CFU/g; *sap2*Δ/Δ *als3*Δ/Δ: 2.72 × 10^5^ CFU/g) (Fig. 3E). In contrast, infection with *ece1*Δ/Δ *hgc1*Δ/Δ resulted in a significantly reduced fungal burden with a median value of 8.32 × 10^4^ CFU/g. A significant reduction in CFU was similarly observed following challenge with *als3*Δ/Δ *ece1*Δ/Δ *hgc1*Δ/Δ, *sap2*Δ/Δ *ece1*Δ/Δ *hgc1*Δ/Δ, and *sap2*Δ/Δ *als3*Δ/Δ *ece1*Δ/Δ *hgc1*Δ/Δ compared to WT (WT: 3.65 × 10^5^ CFU/g; *als3*Δ/Δ *ece1*Δ/Δ *hgc1*Δ/Δ: 3.41 × 10^4^ CFU/g; *sap2*Δ/Δ *ece1*Δ/Δ *hgc1*Δ/Δ: 3.49 × 10^4^ CFU/g; and *sap2*Δ/Δ *als3*Δ/Δ *ece1*Δ/Δ *hgc1*Δ/Δ: 0 CFU/g) (Fig. 3E).

Overall, the day 2 data set was in alignment with the day 3–5 data set and revealed that with additive gene deletions, tongue fungal burden was reduced. Specifically, the combination of hypha formation and candidalysin secretion resulted in high fungal burdens, with limited contribution from Als3p and Sap2p.

### Hyphal growth and candidalysin are required for immune activation in a murine model of OPC

During mucosal infection, *C. albicans* activates the innate immune system. Therefore, to determine whether the selected panel of strains induced differing levels of immune activation, the expression of five target genes induced during *C. albicans* infections (*CXCL1*, *IL-6*, *IL-17A, S100A9,* and *TNF*) was assessed in the tongue by RT-qPCR. Mean gene expression values (relative to a naive control) are presented in Tables S1 and S2.

The immune gene expression data from the initial OPC studies were obtained from mice that succumbed to infection on different days (between days 3 and 5) (Fig. S7A). Therefore, to obtain a more comparable gene expression data set, the experiment was repeated on day 2, with the selected panel of mutant strains.

Compared to WT-infected mice, infection with *ece1*Δ/Δ and *hgc1*Δ/Δ strains led to lower immune activation, which was observed across all gene readouts (*ece1*Δ/Δ: *CXCL1*: 6-fold decrease; *IL-6*: 2-fold decrease; *IL-17A*: 115-fold decrease; *S100A9*: 30-fold decrease; and *TNF*: 5-fold decrease) (*hgc1*Δ/Δ: *CXCL1*: 19-fold decrease; *IL-6*: 14-fold decrease; *IL-17A*: 19-fold decrease; *S100A9*: 59-fold decrease; and *TNF*: 15-fold decrease) (Fig. 3F). This gene expression profile was not observed in response to infection with *als3*Δ/Δ and *sap2*Δ/Δ, which induced immune activation comparable to the WT group. Similarly, infection with the *sap2*Δ/Δ *als3*Δ/Δ double mutant also induced immune activation comparable to the WT group, while infection with all other double, triple, and quadruple mutants caused significantly less immune activation (Fig. 3F).

These data suggest that additive *HGC1* and *ECE1* deletion significantly reduces immune responses. No detectable differences were observed between the naive control and the *sap2*Δ/Δ *ece1*Δ/Δ *hgc1*Δ/Δ-treated mice across all four genes, indicating that this strain is unable to elicit a mucosal immune response. This lack of immune induction was similarly observed with all other strains containing *ECE1* and *HGC1* deletions, suggesting that hyphal growth and candidalysin production are critical for activating a mucosal immune response *in vivo*.

### Hyphal growth and candidalysin are required for mortality in a murine model of OPC

**Fig 4:**
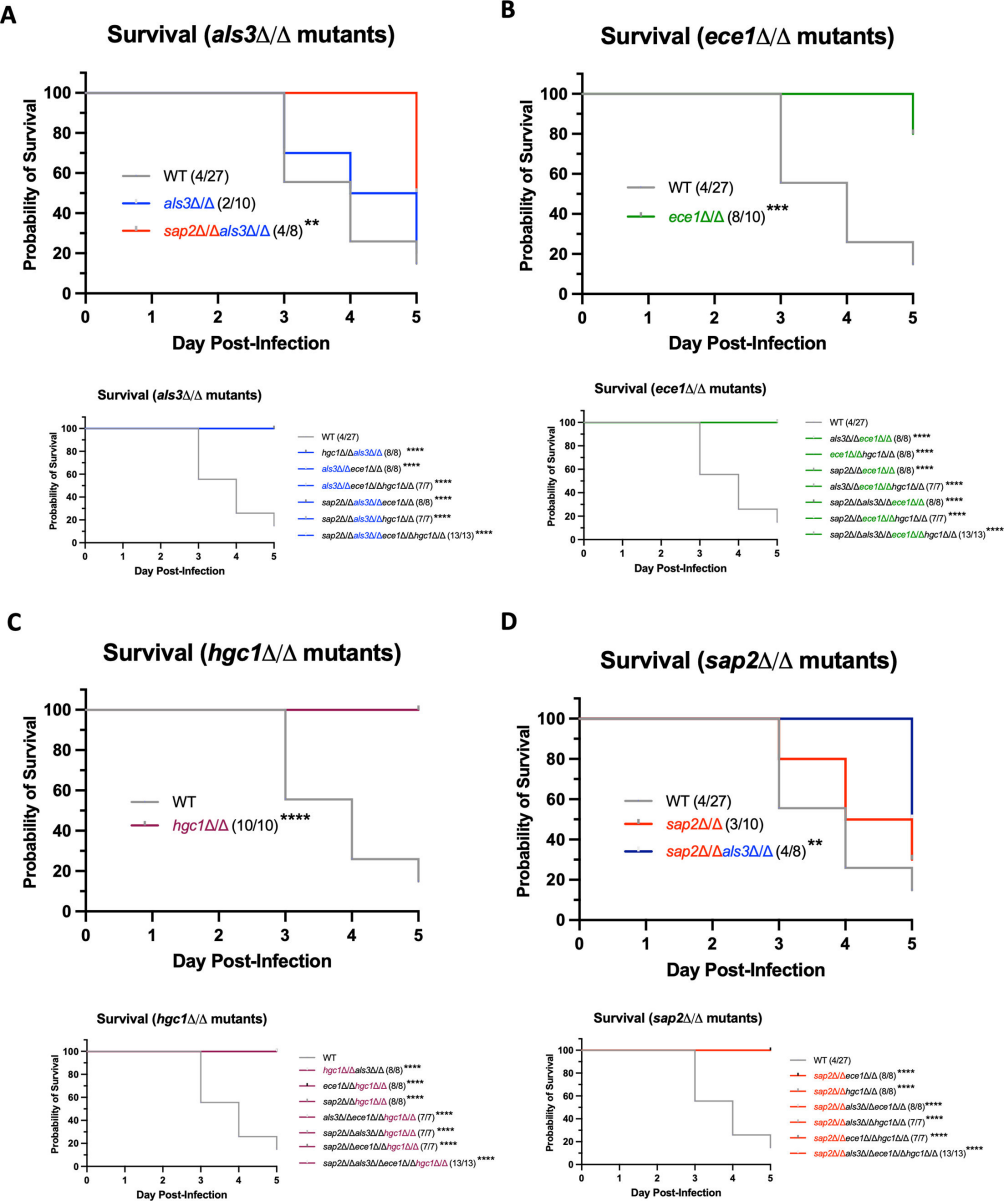
*C. albicans HGC1* and *ECE1* are required for pathogenicity in an immunosuppressed model of OPC infection. Immunocompromised mice were infected for 75 min using a sublingual swab soaked in a 1 × 10^7^ cells/mL solution of *C. albicans* strains and were monitored for survival (based on weight loss or high distress scoring) over 5 days with a total of three doses of cortisone acetate to maintain immunosuppression. Data are separated into infection with (**A**) *als3*Δ/Δ, (**B**) *ece1*Δ/Δ, (**C**) *hgc1*Δ/Δ, and (**D**) *sap2*Δ/Δ single and multi-gene deletion mutants compared to WT (AHY940). The top survival curves depict the respective single deletion mutants and any other multi-deletion mutant that resulted in <100% survival. The bottom survival curves depict all other deletion mutants assessed, resulting in 100% survival. Data are presented as a pooled survival curve (*n* = 8–10 mice per group over two biological repeats) in which a vertical line depicts death. Statistical significance was calculated compared to AHY940 (WT) using a Kaplan-Meier statistical test with a Log-rank (Mantel-Cox) comparison test applied. ****P < 0.0001; ***P < 0.001; and **P < 0.01.

Survival was next assessed once experimental limits were reached (Fig. S7B through E) (27) following challenge with mutant strains in an OPC model. Infection with the WT strain, AHY940, resulted in high mortality, with only a 15% survival rate 5 days after challenge (Fig. 4A through D). The deletion of *ALS3* had no significant effect on survival compared to WT-challenged mice (20% survival) (Fig. 4A). This was also observed following the deletion of *SAP2*, which resulted in a survival rate of 30% (Fig. 4D). In contrast, challenge with the *ece1*Δ/Δ and *hgc1*Δ/Δ mutants resulted in a significant increase in survival: 80% and 100%, respectively (Fig. 4B and C). Interestingly, while *als3*Δ/Δ and *sap2*Δ/Δ single mutant strains had no effect on survival, challenge with the *sap2*Δ/Δ *als3*Δ/Δ double mutant resulted in a survival rate of 50%, significantly higher than the WT group (15%) (Fig. 4A and D). Challenge with all the other double deletion mutants resulted in 100% survival rates (Fig. 4). Challenge with all the triple deletion mutants and quadruple mutants similarly resulted in a 100% survival rate by day 5 post-infection (Fig. 4). These data indicate that hyphal growth and candidalysin secretion are critical for causing mortality in the immunocompromised OPC model, whereas Als3p and Sap2p appear to play a limited role.

## DISCUSSION

*C. albicans* causes over 150 million mucosal infections per year (28), and numerous studies have attempted to identify the critical virulence attributes that are responsible for driving infection. Among these, Als3p, Ece1p, Hgc1p, and Sap2p have been identified as being central factors for pathogenicity, and numerous gene knockout studies have investigated their individual function. However, accurate comparison of host (and fungal) phenotypes is challenging as single gene knockouts are often generated in diverse clinical isolates and strains with different genetic backgrounds. Moreover, while undoubtedly informative, these studies highlight that multiple virulence factors and related attributes are required for successful infection, but our understanding of the interplay between these key factors remains limited.

In this study, we mitigated these issues by using CRISPR-Cas9 gene editing, which has revolutionized the field of *C. albicans* genetic research. It enables the rapid construction of marker-less homozygous deletion mutants and allows multiple deletions in the same strain, facilitating detailed strain comparisons (15). We created a panel of single, double, triple, and quadruple homozygous gene knockouts in a single genetic background to assess the combinatorial roles of Als3p, Ece1p, Hgc1p, and Sap2p during infection. Data revealed that *in vitro,* Als3p, Hgc1p, and candidalysin all play important roles during infection. However, *in vivo*, Hgc1p (thus hyphal growth) and candidalysin together are consistently the most important virulence attributes for driving mucosal infection.

Single deletion of *ALS3*, *ECE1*, *HGC1,* and *SAP2* has been reported previously (4, 8, 24, 29, 30), and our study largely complemented previous phenotypic observations. The impairment of hyphal growth and adhesion/invasion following *HGC1* and *ALS3* deletion, respectively, was conserved across all *hgc1*∆/∆ and *als3*∆/∆ multi-deletion mutants, indicating that additive deletion did not result in phenotypic compensation. Despite previous studies reporting a reduction in adhesion following *HGC1* deletion (31, 32), this was not observed in our assays, indicating Als3p plays a dominant role in epithelial adhesion over hyphal growth (29, 33, 34). This highlights that the use of different genetic modification tools and background strains may impact downstream phenotype, further demonstrating the benefit of re-characterizing mutant strains constructed in one genetic background. Interestingly, we observed an adhesion defect in the *sap2*Δ/Δ *ece1*Δ/Δ *hgc1*Δ/Δ triple mutant, despite the respective single deletion mutants having WT-like adhesion. This indicates a cumulative loss of adherence fitness with multiple deletions and suggests numerous fungal proteins may be indirectly affecting epithelial adhesion.

Invasion assays revealed that Als3p was the primary protein required for invasion into TR146 cells (24, 35, 36), with a lesser but nevertheless observable role for Hgc1p. Candidalysin and Sap2p had no effect, which contrasts with previous studies that reported a link between *SAP1-3*/*SAP4-6* and both induced endocytosis and active penetration invasion routes (37, 38). It is plausible that different Sap family members possess site-specific roles in invasion, but individual roles likely vary.

Despite playing no role in epithelial invasion, candidalysin’s ability to elicit cellular damage and activate immune responses was demonstrated following infection with *ece1*∆/∆ (7–9, 26, 39) and all *ece1*∆/∆ multi-deletion mutants. Adhesion (via Als3p [24, 29, 32, 40]) and hyphal growth (via Hgc1p [32]) have been previously linked to damage. Contrastingly, we observed no differences in damage or immune activation following infection with *hgc1*Δ/Δ due to maintained expression of hypha-associated genes such as *ECE1* (4). This further exemplifies the phenotypic variation between mutant strain backgrounds (BWP17 [32] vs AHY940) and highlights the importance of candidalysin secretion for cellular damage and immune activation, regardless of impaired hyphal growth and invasion. Interestingly, despite this, the double *sap2*Δ/Δ *hgc1*Δ/Δ strain caused significantly less damage than WT, which could be attributable to a cumulative reduction in damage that only gains significance when combined. In support of this, diminished immune activation was observed following infection with all other multi-deletion mutants (not limited to *ece1*∆/∆ strains) in which additive deletions render the mutant less capable of eliciting a potent cytokine response *in vitro*. This suggests that Als3p (40), and to a lesser extent Hgc1p and Sap2p, may be playing a combined role in activating immune responses, driven by candidalysin. We hypothesize that this is due to both a lack of Als3p and Hgc1p-mediated invasion pocket formation and non-efficient candidalysin delivery (7), as well as limited degradation of protective host immune molecules and the extracellular matrix (Sap2p) (13, 41).

To further establish the cooperative interactions revealed from *in vitro* data sets, mucosal *in vivo* modeling was utilized. Oral fungal burden and host immune responses were used as virulence readouts for this model. Additionally, due to the severity of the immunosuppression coupled with infection, we were also able to monitor survival (as mice reached experimental limits). Data revealed that the combination of hyphal growth and candidalysin (via dual *HGC1* and *ECE1* deletion) was vital for establishing OPC infection, culminating in significantly lower fungal burdens in the tongue than the WT strain or the respective single mutants. Accordingly, the challenge with the *ece1*Δ/Δ *hgc1*Δ/Δ strain also did not activate immune cytokine responses and resulted in a 100% survival rate, demonstrating that hyphal growth and candidalysin production together also drive immune activation in the OPC model. Additional deletion of *ALS3* and *SAP2* in the *ece1*Δ/Δ and *hgc1*Δ/Δ background, and the individual *als3*Δ/Δ and *sap2*Δ/Δ mutants, had no effect on fungal burden or immune responses, suggesting *ALS3* and *SAP2* were largely redundant for establishing OPC infection. Candidalysin’s role in promoting OPC infection has been reported previously (8, 39, 42), and Hgc1p has also been linked to OPC infection, in which challenge with *hgc1*Δ/Δ mutants resulted in reduced tongue fungal burden on day 5 (43). Conversely, our study observed no differences following challenge with WT and *ece1*Δ/Δ or *hgc1*Δ/Δ single deletion mutants on day 2 or 5 post-infection, which may reflect differences in mutant construction.

The role of Als3p during OPC has been previously reported, in which challenge with an *als3*Δ/Δ strain led to lower tongue fungal burden on day 1 compared to WT, but this difference had resolved by days 2 (42) and 5 (44). While our study indicated no significant reduction in fungal burdens with *als3*Δ/Δ, there was a trend for reduced CFU on day 2, with any differences resolved by day 5. Challenge with an *als1*Δ/Δ *als3*Δ/Δ double mutant significantly reduced mucosal fungal burden compared to WT (42), suggesting that multiple adhesin proteins are required for adhesion/invasion and thus infection. Thus, our data broadly align with the literature and suggest that while Als3p promotes adhesion to epithelial cells *in vitro* (33), the protein plays a minor role in driving disease in the OPC model.

Elevated *SAP2* gene expression has been observed in *C. albicans* isolated from the oral cavities of humans (carriers and patients with oral candidiasis) (11, 12). However, despite this association, our study found no significant differences following infection between WT and *sap2*Δ/Δ, suggesting that its individual role at the oral mucosa may be limited. Interestingly, combined *ALS3* and *SAP2* deletion led to a significant increase in survival compared to WT-infected mice, supporting the hypothesis that these two gene deletions have a cumulative effect on pathogenicity. As Als3p and Sap2p play a role in invasion (6, 45), their combined action could synergize to promote pathogenicity. This also raises the possibility of genetic compensation occurring in response to singular deletions that may mask virulence defects (32). Despite compensation not being observed *in vitro*, further transcriptomic analysis would be required to establish this *in vivo*.

**Fig 5:**
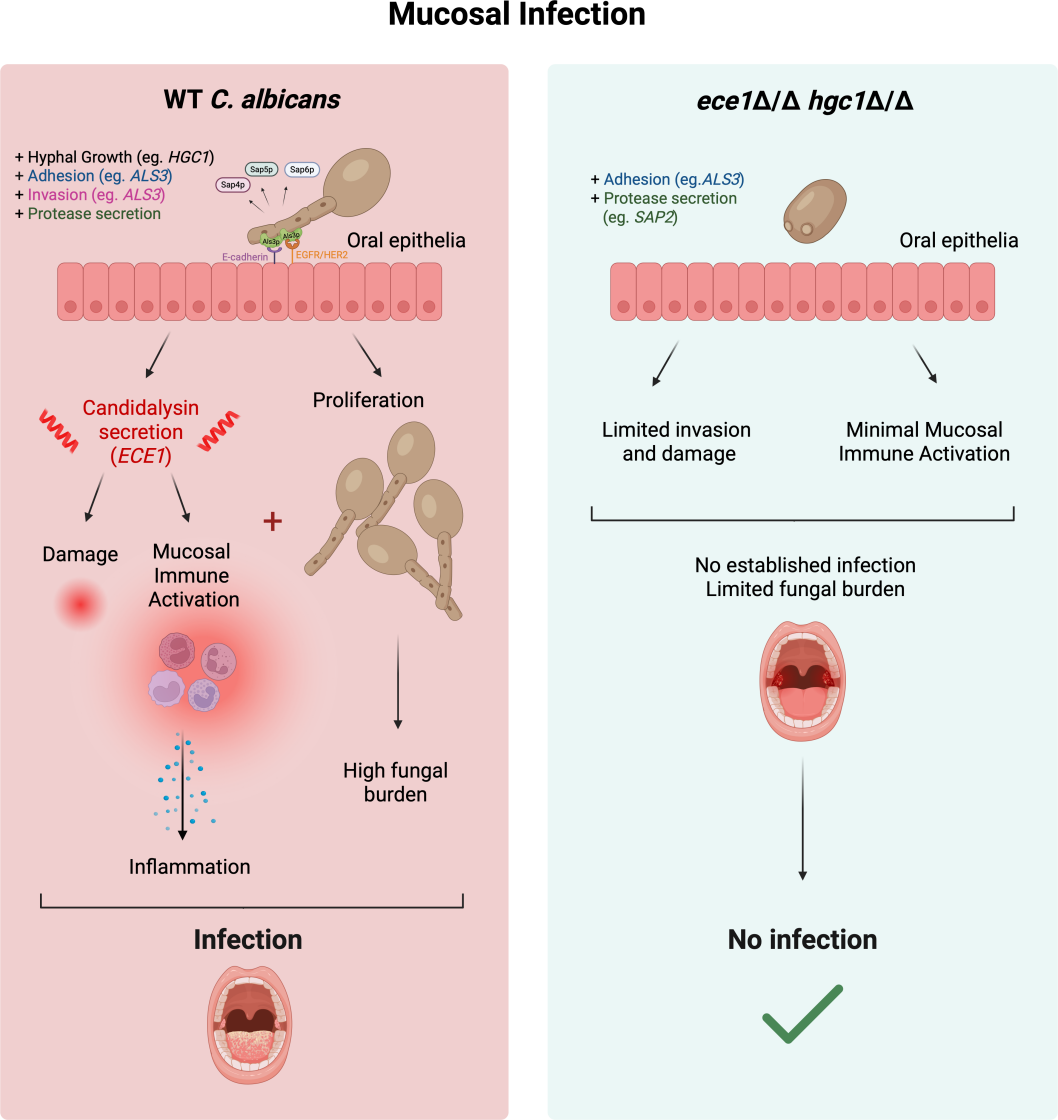
Hypha formation and candidalysin are critical drivers of oral candidiasis. Left (red), infection with WT *C. albicans* leads to hypha formation (e.g., *HGC1*) (aiding Als3p-mediated adhesion and invasion [e.g., *ALS3*]), candidalysin (*ECE1*), and protease secretion. Specifically, the dual action of hyphal growth and candidalysin results in tissue damage and the subsequent release of pro-inflammatory cytokines and the activation of mucosal innate immune responses. WT *C. albicans* can also proliferate, leading to high oral fungal burdens. Overall, this leads to infection and often mortality in the immunocompromised murine OPC infection model. Right (blue), infection with *ece1*Δ/Δ *hgc1*Δ/Δ, which neither forms hyphae nor secretes candidalysin (but can still express hypha-associated genes, e.g., *ALS3*), fails to damage the epithelium or activate the innate immune system (despite adhesion and protease secretion [e.g., *SAP2*]), and *C. albicans* cannot establish infection, as highlighted by low tongue fungal burden. Figure was created with BioRender.

Overall, our *in vivo* data sets reveal a crucial relationship between hyphal growth (via *HGC1*) and candidalysin, with minimal impact from Als3p and Sap2p. Our data support a hypothesis that without hyphal growth (via *HGC1* deletion), epithelial invasion is highly compromised. This prevents the formation of the invasion pocket and the subsequent delivery of candidalysin that drives damage, epithelial signaling, and immune activation, thus establishing an infection phenotype (Fig. 5). Therefore, both hyphal growth and candidalysin secretion act in a synergistic manner to promote pathogenicity.

It must be noted that during the construction of our mutant strains, it was discovered that AHY940, the parental WT strain, was potentially trisomic for Chromosome 5 (46). This may lead to the overexpression of specific proteins that could influence cell wall architecture (e.g., *PGA4* and *CHT2* [47]), drug susceptibility (48), host interactions, or virulence (47, 49, 50). Chromosome 5 trisomy has been previously linked to a commensal-like phenotype in *C. albicans* during oropharyngeal candidiasis (50), although this was not observed during our murine experiments. Trisomy can also lead to an unstable genome, increasing the likelihood of recombination errors and spontaneous loss of heterozygosity, which can further influence phenotype (51). Nevertheless, aneuploidy can spontaneously resolve (52), which has been previously reported during mutant construction using AHY940 (46), suggesting that the WT strain or some of the strains constructed in this study may not have retained Chromosome 5 trisomy. Although outside the scope of this study, karyotyping analysis would be useful to establish the possible impact of Chromosome 5 trisomy on our findings.

In summary, this study used CRISPR-Cas9 and homology-directed repair technology to construct homozygous gene knockouts of *ALS3*, *ECE1*, *HGC1,* and *SAP2* in *C. albicans*, both individually and in combination, to investigate their combinatorial roles in the context of infection. The panel of 19 mutant strains was initially characterized *in vitro*, revealing attenuated virulence traits following singular gene deletions; however, additive deletions often led to cumulative phenotypic impairments. *In vivo*, during OPC, the key drivers of infection were hyphal growth and secretion of candidalysin, exemplified by the double *ece1*Δ/Δ *hgc1*Δ/Δ mutant causing significantly impaired tongue fungal burden and immune activation, alongside reduced mortality. These data provide critical insights into the combinatorial roles of virulence attributes in the development of mucosal *C. albicans* infection. This work highlights the importance of viewing virulence genes not as separate entities, but as components of a collective network, acting together to promote pathogenicity. This observation may also extend to other fungal species, as intracellular cooperation has been reported in the fungal plant pathogen *Sclerotinia sclerotiorum*, which utilizes synergistic division of labor and resource allocation to increase invasive growth (53). Our work, therefore, not only enhances our fundamental understanding of fungal pathogenesis but also lays the groundwork for the improved development of targeted treatment strategies.

## MATERIALS AND METHODS

### *Candida albicans* strains and growth conditions

The *C. albicans* strains used in this study are presented in Table 1. The *C. albicans* wild-type strain AHY940 is an isogenic descendant of SC5314 except for a heterozygous deletion of the *LEU2* ORF to facilitate the integration of CRISPR components (15). Additionally, this strain has a possible trisomy of Chromosome 5 (48). All strains used in this study were initially grown at 30°C on yeast extract-peptone-dextrose agar containing 1% yeast extract, 2% peptone, 2% dextrose, and 1.5% agar. Overnight cultures were grown for 16 h in YPD broth at 180 RPM and 30°C, unless stated otherwise.

**TABLE 1:** *C. albicans* strains used in this study

Strain name	Description	Genotype	Source
AHY940	SC5314 *LEU2* heterozygous knockout (possible trisomy of Chromosome 5)	a/α *leu2*Δ/*LEU2*	(15)
OP01	*als3*Δ/Δ	*leu2*Δ/*LEU2 als3*Δ/Δ	This study
OP02	*ece1*Δ/Δ	*leu2*Δ/*LEU2 ece1*Δ/Δ	This study
OP03	*hgc1*Δ/Δ	*leu2*Δ/*LEU2 hgc1*Δ/Δ	This study
OP04	*sap2*Δ/Δ	*leu2*Δ/*LEU2 sap2*Δ/Δ	This study
OP05	*als3*Δ/Δ *ece1*Δ/Δ	*leu2*Δ/*LEU2 als3*Δ/Δ *ece1*Δ/Δ	This study
OP06	*hgc1*Δ/Δ *als3*Δ/Δ	*leu2*Δ/*LEU2 hgc1*Δ/Δ *als3*Δ/Δ	This study
OP07	*sap2*Δ/Δ *als3*Δ/Δ	*leu2*Δ/*LEU2 sap2*Δ/Δ *als3*Δ/Δ	This study
OP08	*ece1*Δ/Δ *hgc1*Δ/Δ	*leu2*Δ/*LEU2 ece1*Δ/Δ *hgc1*Δ/Δ	This study
OP09	*sap2*Δ/Δ *ece1*Δ/Δ	*leu2*Δ/*LEU2 sap2*Δ/Δ *ece1*Δ/Δ	This study
OP10	*sap2*Δ/Δ *hgc1*Δ/Δ	*leu2*Δ/*LEU2 sap2*Δ/Δ *hgc1*Δ/Δ	This study
OP11	*als3*Δ/Δ *ece1*Δ/Δ *hgc1*Δ/Δ	*leu2*Δ/*LEU2 als3*Δ/Δ *ece1*Δ/Δ *hgc1*Δ/Δ	This study
OP12	*sap2*Δ/Δ *als3*Δ/Δ *ece1*Δ/Δ	*leu2*Δ/*LEU2 sap2*Δ/Δ *als3*Δ/Δ *ece1*Δ/Δ	This study
OP13	*sap2*Δ/Δ *als3*Δ/Δ *hgc1*Δ/Δ	*leu2*Δ/*LEU2 sap2*Δ/Δ *als3*Δ/Δ *hgc1*Δ/Δ	This study
OP14	*sap2*Δ/Δ *ece1*Δ/Δ *hgc1*Δ/Δ	*leu2*Δ/*LEU2 sap2*Δ/Δ *ece1*Δ/Δ *hgc1*Δ/Δ	This study
OP15	*sap2*Δ/Δ *als3*Δ/Δ *ece1*Δ/Δ *hgc1*Δ/Δ	*leu2*Δ/*LEU2 sap2*Δ/Δ *als3*Δ/Δ *ece1*Δ/Δ *hgc1*Δ/Δ	This study
OP16	*als3*Δ/Δ + *ALS3*	*leu2*Δ/*LEU2 als3*Δ/Δ + *ALS3*	This study
OP17	*ece1*Δ/Δ + *ECE1*	*leu2*Δ/*LEU2 ece1*Δ/Δ + *ECE1*	This study
OP18	*hgc1*Δ/Δ + *HGC1*	*leu2*Δ/*LEU2 hgc1*Δ/Δ + *HGC1*	This study
OP19	*sap2*Δ/Δ + *SAP2*	*leu2*Δ/*LEU2*:*NAT1 sap2*Δ/Δ + *SAP2*	This study

### Mammalian cell culture

All cell culture experiments were performed using the TR146 human buccal epithelial squamous cell carcinoma cell line, which was purchased from the European Collection of Authenticated Cell Cultures (ECACC 10032305) (54).

Cells were cultured in Dulbecco’s modified Eagle’s medium (DMEM/F-12, Gibco) supplemented with 15% fetal bovine serum, 50 µg/mL streptomycin, and 100 µg/mL penicillin (Sigma-Aldrich) in 5% CO_2_ at 37°C. Mycoplasma testing was carried out routinely by PCR. Cells were passaged at 70%–90% confluence. For experimentation, cells were seeded at a concentration of 5 × 10^5^/mL in 12- or 24-well plates and grown at 37°C with 5% CO_2_ for 24 h until 90%–100% confluent. After 24 h, cells were serum-starved with fresh serum-free medium. The following day, cells were infected with *C. albicans* strains in serum-free medium or with PBS as a control. Cultures were washed twice in Dulbecco’s phosphate-buffered saline (Gibco), and absorbance was measured to determine fungal cell density prior to infection. A multiplicity of infection of 10, 1, and 0.01 was chosen for 2, 4, and 24 h time points, respectively. After infection, plates were incubated at 37°C in a humidified incubator with 5% CO_2_ for their designated time points.

### Plasmids for CRISPR-Cas9 transformation

All CRISPR protocols were based on previously established methods developed by the Hernday laboratory (15) using pADH110 (Addgene, #90982), pADH119 (Addgene, #90985), and pADH137 (Addgene, #90986) (15). Prior to transformation, 2 µg of pADH137 DNA was digested with *Mss*I (Thermo Fisher, #ER1341) for approximately 1 h to linearize the plasmid and facilitate Cas9-induced DNA cutting.

### CRISPR design

gRNAs for *ALS3*, *ECE1*, *HGC1,* and *SAP2* were created using the Design and Analyze Guides tool on Benchling (benchling.com) with the following parameters: design type: single guide; guide length: 20 bp; genome: CA22 (*C. albicans* SC5314); PAM: NGG. After identification, flanking sequences were added (5′-CGTAAACTATTTTTAATTTG[20 bp target sequence]GTTTTAGAGCTAGAA) to facilitate gRNA stitching. All sequences are listed in Table S3. Repair templates/donor DNA (dDNA) were designed with the intention of removing the whole open reading frame of target genes. The addition of a GG or computer-generated sequence (designed via the ADD-TAG software [55]) in place of the ORF was used to create a new PAM site for future gene reintegration studies. For *SAP2*, dDNA was designed to introduce a stop codon (TAA), a unique endonuclease restriction site (*Sap*I), and GG.

### Pre-transformation polymerase chain reactions

#### dDNA annealing

dDNA was prepared by annealing 100 µM of sense and complementary oligonucleotides and reducing the reaction temperature from 99°C to 65°C at a rate of 1°C/min in a Veriti 96-well Thermal Cycler (Applied Biosystems).

#### gRNA stitching

gRNA fragments A and B were stitched together to form a Fragment C gRNA expression cassette (used for transformation) in a Veriti 96-well Thermal Cycler (Applied Biosystems) using Phusion polymerase (Thermo Fisher, #F530S). Fragment A was generated via PCR using pADH110 as the DNA template for oligos AHO1096 and AHO1097. Fragment B was generated using pADH119 as the DNA template and custom gRNA and AHO1097 oligos. Finally, these two fragments were stitched together using amplification oligos AHO1237 and AHO1238. PCR cycling conditions and reagent concentrations are described in Table S4.

### Lithium acetate transformation of *Candida albicans*

Overnight cultures were diluted 1:50 in YPD and incubated for approximately 2 h until an absorbance (600 nm) of 0.5–0.8 was reached, after which cells were pelleted, washed, and resuspended in 1/100 of the original volume. In an Eppendorf tube, 50 µL of the fungal cell suspension was added to 10 µL dDNA, 2 µg *Mss*I-digested Cas9 plasmid (pADH137), 50 µL gRNA fragment C, 1 mL PLATE mix (875 µL 50% PEG 3350 [Sigma-Aldrich], 100 µL TE [100 mM Tris-HCl, pH 7.4, 10 mM ethylenediaminetetraacetic acid {EDTA}, pH 8.0] [Sigma-Aldrich], 25 µL 1 M lithium acetate, pH 7.0 [MP Biochemicals, #02155256-CF]), and 10 µL 10 mg/mL boiled and snap cooled salmon sperm DNA (Thermo Fisher, #15632011). Samples were mixed by inversion and gentle flicking and were incubated statically overnight at 30°C. Fungal cells were then heat shocked at 44°C for 15 min, then pelleted and washed twice in YPD medium. The pellet was resuspended in 1 mL YPD and added to an additional 1 mL YPD in a 15 mL Falcon tube, and the suspension was incubated at 30°C, 200 RPM for 5 h. Following incubation, samples were pelleted and re-suspended in the residual liquid and plated onto YPD agar containing 200 µg/mL nourseothricin (Jena Bioscience, #AB-102XL) for positive selection. Plates were left to grow at room temperature for 3 days.

Excision of the CRISPR components was achieved by growing transformants on synthetic defined (SD) agar medium lacking leucine at 30°C in a static incubator and patching single colonies onto YPD and YPD + 400 µg/mL nourseothricin for confirmation of the loss of the antibiotic resistance marker.

### Extraction of fungal genomic DNA

Extraction of genomic DNA (gDNA) from *C. albicans* was performed by growing an overnight culture to the stationary phase in YPD medium at 30°C and 200 RPM. Cultures were harvested by centrifugation, and pellets were washed twice with sterile water. The pellet was then resuspended in 0.2 mL solution A (2% Triton X-100, 1% sodium dodecyl sulfate [SDS], 100 mM NaCl, 10 mM Tris-HCl [pH 8.0], and 1 mM Na_2_EDTA) and transferred into a 2 mL screw cap tube containing one-third volume of 0.5 mm glass beads. In a fume hood, 0.2 mL phenol/chloroform/isoamyl alcohol (25:24:1 volumetric ratio, Thermo Fisher, #15593049) was added, and samples were bead-bashed using a Fast Prep 24 5G disruptor (MP Biomedicals) at 2.5 m/s for 30 s three times, resting the samples for 2 min on ice between each cycle. Following lysis, 0.2 mL of TE buffer (10 mM Tris-HCl, pH 8.0, and 1 mM EDTA) was added, and samples were centrifuged for 5 min at 17,000 × *g* at 4°C. The aqueous upper layer was removed and added to 1 mL of 100% ethanol and chilled at −20°C for 30 min. The samples were then centrifuged at 17,000 × *g* for 10 min at 4°C, and the pellet was resuspended in 0.4 mL TE buffer with 1.5 µL of RNase A solution (20 mg/mL) (New England Biolabs, #T3018L). Samples were incubated at 37°C for 5 min, and DNA was precipitated by adding 10 µL of 3 M sodium acetate (pH 5.2) and 1 mL of 100% ethanol before being centrifuged at 17,000 × *g* for 10 min at 4°C. The supernatant was discarded, and pellets were washed with 100 µL of 70% ethanol. Pellets were left to air dry before being resuspended in sterile water. The concentration of gDNA was quantified using a NanoDrop 2000 spectrophotometer (Thermo Fisher).

### Colony PCR

Primers were designed using Primer3Plus software (bioinformatics.nl/cgi-bin/primer3plus/primer3plus.cgi) with the following parameters: length: 18–25 bp; GC content: 35%–60%; annealing temperature: 57°C–64°C; GC clamp: 1. All oligonucleotides used in this project are listed in Table S3.

A small portion of each colony was transferred into a PCR tube containing buffer, deoxyribonucleotide triphosphate (dNTPs), and primers and heated to 99°C in a thermocycler with a heated lid for 10 min (see Table S5 for reagent concentrations and cycler conditions). After completion, Hot Start II Phire polymerase (Thermo Fisher, #F122S) was added to each tube, and PCR amplification was performed (Table S5). PCRs were electrophoresed at 120 V for approximately 20 min on a 1% agarose gel supplemented with 2 µL Gel Red (MedChemExpress, #HY-K1007). Gels were imaged using an Odyssey Fc Imaging System (LI-COR). Following a positive cPCR result (indicating a gene knockout), relevant colonies were re-streaked onto YPD agar supplemented with 200 µg/mL nourseothricin and grown at 30°C. Overnight cultures of the re-streaked colony were then prepared for the extraction of genomic DNA by culturing in YPD medium supplemented with 200 µg/mL nourseothricin. PCRs were then performed using approximately 50 ng of gDNA, with Phusion 5× HF buffer (Thermo Fisher), dNTPs, flanking primers, and Phusion polymerase (Thermo Fisher). See Table S6 for reagent concentrations and cycler conditions. Reactions were electrophoresed at 120 V for approximately 20 min on a 1% agarose gel supplemented with 2 µL Gel Red. Gels were either imaged using an Odyssey Fc Imaging System (LI-COR) or DNA was excised from the gel for sequencing.

### DNA sequencing

Isolated fungal gDNA was PCR amplified using flanking primers, and 50 µL of the PCR product was electrophoresed at 120 V for approximately 20 min on a 1% agarose gel supplemented with 2 µL of Gel Red (MedChemExpress). DNA bands were visualized using a MyView Compact UV Transilluminator (Accuris) and excised from the gel. DNA was extracted using a QIAquick Gel Extraction Kit (Qiagen, #28704) according to the manufacturer’s instructions. Purified samples were analyzed by DNA sequencing (Eurofins; Source BioScience; BioBasic) using the forward flanking primer (sample concentration 1–10 ng/µL; primer concentration 10 pmol/µL [see Table S3 for oligonucleotides used]).

### RNA isolation from *Candida albicans*

*C. albicans* strains were grown overnight in YPD at 30°C and 200 RPM. Cells were harvested by centrifugation, and the pellet was washed twice in PBS. For each strain, two diluted cultures were prepared: one in YPD and one in Roswell Park Memorial Institute medium + 10% FBS. Cultures were placed back in the shaking incubator for 2.5 or 3 h (experiment dependent) at 30°C and 37°C, respectively. Following incubation, cultures were centrifuged at 2,500 × *g* for 3 min at 4°C, and the exhausted culture medium was removed. The remaining pellet was flash-frozen in liquid nitrogen before being resuspended in 400 µL AE buffer (50 mM sodium acetate, pH 5.2, and 10 mM EDTA). In a fume hood, 37.5 µL of 25% SDS was added to each sample with 400 µL of phenol (pH 4.3). Samples were incubated at 65°C for 20 min and vortexed every minute, then placed on ice for 5 min. Next, samples were microcentrifuged at 17,000 × *g* for 15 min at 4°C, and the supernatant was transferred into a clean tube. To this, 500 µL of 100% chloroform was added, and samples were mixed by vortexing. Each tube was microcentrifuged at 10,000 × *g* for 15 min at 4°C, and the supernatant was transferred into a new tube. A volume of 400 µL of chloroform was added to the supernatant before being vortexed and microcentrifuged at 10,000 × *g* for 15 min at 4°C. The supernatant was removed (200 µL), and 20 µL of 3 M sodium acetate was added with 200 µL of 100% isopropanol. Tubes were gently inverted to precipitate RNA and incubated at 4°C for 1 h. Samples were microcentrifuged at 17,000 × *g* for 20 min at 4°C before removing the supernatant. Pellets were washed with 1 mL of 70% RNase-free ethanol and re-microcentrifuged at 17,000 × *g* for 20 min at 4°C. The supernatant was removed, and the pellets were air-dried before being re-suspended in 20–50 µL of RNase-free water. Dilutions in water (1:10) were prepared from the stock solution, and the diluted RNA concentration was quantified using a NanoDrop 2000 spectrophotometer (Thermo Fisher).

### Reverse transcription quantitative PCR analysis

RNA (2 µg) was converted to cDNA as per the manufacturer’s instructions using a High-Capacity cDNA Reverse Transcriptase Kit (Thermo Fisher, #4368814). Samples were prepared for reverse transcription quantitative real-time PCR using 100 ng of cDNA, 1× SYBR Green (Qiagen, #208056), and 500 nM of relevant primers (Table S3). RT-qPCR was performed in a 384-well plate on a QuantStudio 7 Flex Real-Time PCR System, and gene expression was calculated using the threshold cycle (ΔΔ*C_T_*) method, using actin as the housekeeping gene and yeast-morphology WT *C. albicans* (AHY940) as the control.

### BSA digestion assay for Sap2p activity

Strains were grown overnight, cells were washed twice in PBS, and absorbance was measured. Cultures were prepared in Yeast Carbon Base (New England Biolabs, #B9017S) + 1% bovine serum albumin (BSA) medium (pH 4.0, Sigma-Aldrich, #A3059) at an absorbance of 0.1 (600 nm). Cultures were then incubated at 30°C (200 RPM), and absorbance was measured daily for 7 days.

### Hyphal morphogenesis assay

AHY940 and mutant strains were grown overnight, and cells were washed twice and counted using a hemocytometer. Concentrations of 1 × 10^7^ cells/mL were prepared in PBS and then diluted to a working concentration of 1 × 10^6^ cells/mL in YPD. A volume of 90 µL of each cell suspension was added to a 96-well plate with the addition of 10 µL filtered FBS (using an Ultra-3k Centrifugal Filter Device [Amicon]) to induce hypha formation. A control plate was prepared by adding 10 µL of PBS to 90 µL of cell suspension. Both plates were covered with material membranes and incubated at 30°C (control) or 37°C (hypha-inducing) and 100 RPM for 3 h. Each well was imaged directly using a ×20 magnification Leica Leitz microscope or fixed with 8 µL of 3.7% formaldehyde for subsequent imaging.

### Epithelial adhesion and invasion assays

Glass coverslips were added to each well of a 24-well polystyrene plate, and the plate was microwaved for 1 min to sterilize coverslips. TR146 cells were then seeded at a concentration of 1 × 10^5^ cells/mL, and 500 µL of this cell suspension was added to each well. Plates were incubated at 37°C + 5% CO_2_ for 24 h, after which the medium was refreshed. After another 24 h, cells were washed once in PBS, and 400 µL of serum-free medium was added to each well. Cells were then infected with 100 µL of pre-washed *C. albicans*, which had been adjusted to a concentration of 1 × 10^6^ cells/mL in serum-free medium (MOI 1). Plates were incubated at 37°C + 5% CO_2_ for 1 h, after which the medium was removed, and cells were washed twice with cold PBS. Next, cells were treated with 300 µL of 5% paraformaldehyde for 30 min at 37°C + 5% CO_2_ before being washed twice with cold PBS and refrigerated overnight with 1 mL of PBS per well. The following day, wells were washed three times with cold PBS before 200 µL of 10 µg/mL Concanavalin-A AlexaFluor 467 solution was added to each well. Plates were protected from light (for all staining steps) and incubated at room temperature for 45 min at 70 RPM. Following incubation, wells were washed a further three times with cold PBS before 300 µL of 0.1% TritonX-100 was added per well to facilitate permeabilization. Plates were incubated at room temperature for 15 min at 70 RPM, before undergoing three additional PBS washes. Cells were then incubated with 500 µL of 10 µg/mL calcofluor white prepared in 100 mM Tris-Cl (pH 9.5) for 20 min at 100 RPM. Finally, plates were washed three times with 1 mL of sterile water, incubating for 5 min at 180 RPM at 30°C for each wash. Following the final wash, water was left in the wells to facilitate the removal of the cover slip. To prepare microscopy slides, 6 µL of Prolong Antifade was pipetted onto each slide, and coverslips were gently mounted using forceps and a pipette tip. Slides were air-dried for 2 h before imaging and stored at -20°C long-term. Images were taken on a Zeiss Apotome Microscope at ×20 magnification to capture a large number of cells per image and calculate overall adhesion/invasion efficiency. The Z-stack function was used to capture the in-focus regions from different planes.

Multiple images from each slide were taken until either a total of at least 60 adherent *C. albicans* cells were counted, or eight images were taken (for non-adherent strains) in each of three biological replicates. The percentage of adherent cells per coverslip was calculated using the following formula:


(1) $$\%\text{ adherence to epithelial cells }=\frac{average\;cell\;count\;in\;the\;square\;\times \;area\;of\;the\;well\;in\;\mu m^{2}}{area\;of\;the\;square\;in\;\mu m^{2}\;\times \;total\;input\;Candida\;in\;each\;well}\times 100.$$

### Invasion

For invasion, the same method was used as described for adhesion, but infections were incubated for 4 h. Data are presented as the percentage of fungal cells invaded into epithelial cells out of the total count of fungal cells per image, with a minimum of three images taken per strain in each of three biological replicates.

### Cell damage assay

Following 24 h incubation, the exhausted culture medium was collected from cell culture plates infected with fungal strains (MOI 0.01) and clarified by centrifugation at 10,000 × *g* for 10 min at 4°C. LDH activity was quantified to determine cell damage using a Cytotox96 Non-Radio Cytotoxicity Assay Kit (Promega, #G1782). A standard curve was created using recombinant porcine LDH (Sigma-Aldrich). Absorbance was measured at 492 nm using an Infinite F50 plate reader (Tecan).

### Extraction of epithelial protein

At the desired time points, the exhausted culture medium was aspirated from the cells, and the cell culture plates were placed on ice. Cells were washed twice with cold PBS prior to the addition of a modified RIPA lysis buffer (50 mM Tris-HCl, pH 7.4, 150 mM NaCl, 1 mM EDTA, 1% Triton X-100, 1% sodium deoxycholate, and 0.1% SDS) supplemented with protease and phosphatase inhibitors (1:100 dilution) (Sigma-Aldrich) to facilitate cell lysis. After a 2-min incubation with lysis buffer, adherent cells were scraped from the plate using a plastic scraper and were transferred into pre-cooled microcentrifuge tubes. Lysate samples were incubated on ice for a minimum of 30 min before being microcentrifuged at 13,000 × *g* at 4°C for 10 min. Supernatants were transferred into clean tubes for protein quantification.

Lysate protein concentrations were quantified using a Microplate Bicinchoninic Acid (BCA) Protein Assay Kit (Thermo Fisher, #23252) according to the manufacturer’s instructions. Briefly, in a 96-well plate, a BSA standard curve was generated. Duplicate samples were diluted in PBS, and BCA working reagent was added to each well (1:50 of reagents B and A). Following a 30-min incubation at 37°C in the dark, the absorbance was measured at 562 nm using an Infinite F50 plate reader (Tecan).

### Western blot sample preparation and SDS-PAGE

Samples were prepared in a total volume of 37.5 µL containing 30 µg protein, Laemmli sodium dodecyl sulfate sample buffer (4×) (Thermo Fisher, #J63615), dithiothreitol (50 mM), and PBS (remaining volume). Samples were heated at 85°C for 8 min before 15 µL of each sample was added to individual wells of a Novex Tris-Glycine Mini Protein Gel (4%–20%, Invitrogen, # XP04200PK2) in an XCell *SureLock* Mini-Cell tank filled with NuPAGE MOPS SDS Running Buffer (Thermo Fisher, #NP0001). The Color Pre-stained Protein Standard, Broad Range (10–250 kDa) (New England BioLabs, #P7719S) ladder was used for molecular weight estimations. Samples were electrophoresed at 100 V for approximately 80 min.

### Western blotting analysis

After electrophoresis, proteins were transferred onto a nitrocellulose membrane using the Quick Transfer 7-min protocol and a TransBlot Turbo Transfer System (BioRad). Membranes were blocked for 1 h in 5% skimmed milk in TBS-T (Tris-buffered saline and 0.1% Tween 20) with shaking and were then washed once with TBS-T. Membranes were then incubated with primary antibodies and gently agitated at 4°C overnight (Table 2). The next morning, membranes were washed six times for 5 min in TBS-T before the addition of the secondary antibody for 1 h at room temperature with agitation (Table 2). Six additional 5-min washes were then performed with TBS-T before protein detection using Immobilon Western Chemiluminescent HRP Substrate (Merck Millipore, #WBKLS0500). Visualization was performed using an Odyssey Fc Imaging System (LI-COR), and densitometry quantification was performed using Image Studio RM Lite (LI-COR Biosciences) software. α-actin was used as a loading control.

**TABLE 2:** Antibodies used for western blotting in this study

Antibody	Dilution	Reconstitution diluent	Species	Company	Product code
Phospho-DUSP1/MKP1 (Ser359) (125E2)	1:1,000	5% BSA	Rabbit	Cell Signaling Technology	35217
c-Fos (9F6)	1:1,000	5% milk	Rabbit	Cell Signaling Technology	2250
α-Actin (clone C4)	1:10,000	5% milk	Mouse	Merck Millipore	MAB1501
Secondary: peroxidase-conjugated AffiniPure goat anti-mouse	1:20,000	5% milk	Goat	Jackson Immunoresearch	115-035-062
Secondary: peroxidase-conjugated AffiniPure goat anti-rabbit	1:20,000	5% milk	Goat	Jackson Immunoresearch	111-035-003

### Quantification of cytokine secretion

Exhausted cell culture medium from infection plates (MOI 0.01) was collected at 24 h and microcentrifuged at 10,000 × *g* for 10 min at 4°C. The concentration of cytokines was determined using magnetic microparticles (R&D Systems) specific for human granulocyte colony-stimulating factor, granulocyte-macrophage colony-stimulating factor, interleukin-1α, IL-1β, and IL-6 cytokines using a magnetic Luminex performance assay (R&D Systems) and a Bio-Plex 200 system (Bio-Rad) according to the manufacturer’s instructions. Data were analyzed using Bioplex Manager 6.1 software.

### Establishment of immunocompromised murine oropharyngeal *Candida albicans* infection model

Sample size was determined using the *R* equation (56). Seven- to eight-week-old male and female C57BL/6 mice (Jackson Laboratory) were used in all experiments. Mice were randomly allocated into different groups and ear clipped for identification prior to experimentation. Mice were injected subcutaneously with cortisone-21-acetate (225 mg/kg of body weight, Sigma-Aldrich, #46149) on the day prior to infection, day 1 post-infection, and day 3 post-infection. Fungal strains were prepared by washing overnight cultures twice in PBS and serially diluting to facilitate counting via hemocytometer. Cell suspensions were prepared at a concentration of 1 × 10^7^ cells/mL in PBS, and sterile swabs were submerged in the solution. On day 0, mice were sedated using 75 mg ketamine/kg of body weight (100 mg/mL, #QN01AX03) and 1 mg medetomidine/kg of body weight (1 mg/mL, #VPA10664/005/001) administered via intraperitoneal injection. Swabs were placed sublingually for 75 min before removal and intraperitoneal administration of atipamezole (5 mg/mL, #QV03AB90) to reverse the anesthesia. Mice were placed on their side to recover and monitored until they were behaving normally. Mice were weighed and scored daily using a distress scoring chart adapted from Lloyd and Wolfensohn (27), and once experimental limits were reached (e.g., ≤80% of their initial weight), mice were euthanized humanely via an intraperitoneal injection of 0.8 mg pentobarbital sodium/kg of body weight (200 mg/mL, #A003028). Experiments were conducted for 5 days, and all mice were then euthanized humanely. Tongues were collected (on the day of death or day 5) and were halved longitudinally, with one half being used for fungal burden quantification and the other for cytokine analysis.

### Oral fungal burden analysis

At days 1, 2, and 5 post-infection, three to four mice per group were sacrificed for quantifying fungal burden in tongue tissue. Tongues were harvested, stored in PBS, and placed on ice before being weighed and transferred into gentleMACS C tubes (Miltenyi Biotec, #130-093-237) containing 1 mL of PBS for homogenization. Tubes were placed in a gentleMACS dissociator (Miltenyi Biotec) and were treated approximately seven times, until homogenized. Tubes were centrifuged at 2,500 × *g* for 1 min at 4°C, and lysate was transferred to an empty microcentrifuge tube. From this, three 1:10 serial dilutions were prepared in PBS, and 20 µL of each cell suspension was plated in duplicate onto YPD agar plates containing 50 µg/mL chloramphenicol. Colonies were counted after incubation at 37°C for 24 h, and fungal burden per gram of tissue was determined. The remaining tissue homogenates were stored at −80°C.

### *In vivo* RT-qPCR cytokine analysis

Cytokine gene expression in the tongue was quantified using RT-qPCR. First, tongues were placed in gentleMACS M tubes (Miltenyi Biotec, #130-093-236) with 600 µL of RLT plus lysis buffer (Qiagen, #74134) and homogenized for 1 min in a gentleMACS dissociator (Miltenyi Biotec). Tubes were then centrifuged twice at room temperature for 3 min at 2,500 × *g* prior to RNA extraction using the RNeasy Plus Mini Kit (Qiagen, #74134), according to the manufacturer’s instructions. RNA was quantified using a NanoDrop 2000 spectrophotometer, and 2 µg was reverse-transcribed to cDNA as per the manufacturer’s instructions using a High Capacity cDNA Reverse Transcriptase Kit (Thermo Fisher, #4368814). Samples were prepared for RT-qPCR using 100 ng of cDNA, 1× SYBR Green (Qiagen, #208056), and 500 nM of relevant primers (Table S3). RT-qPCR was performed in a 384-well plate on a QuantStudio 7 Flex Real-Time PCR System, and gene expression was calculated using the threshold cycle (ΔΔ*C_T_*) method, using *GAPDH* as the housekeeping gene and cDNA from naive mouse tongue as the control.

### Statistical analyses

At least three biological replicates were performed per experiment, except where otherwise stated. Data were analyzed using Prism 10 (GraphPad Software). Statistical analysis used for individual experiments is stated in the figure legends.

## SUPPLEMENTAL MATERIAL

Fig. S1 mbio.03304-25-s0001.pdf Quantification of gene expression in *C. albicans* mutant strains constructed for this study.

Fig. S2 mbio.03304-25-s0002.tif Quantification of *ALS* family gene expression in selected *C. albicans* mutant strains.

Fig. S3 mbio.03304-25-s0003.tif Compensatory changes in *SAP* family expression are not observed in selected mutant strains.

Fig. S4 mbio.03304-25-s0004.pdf *C. albicans als3*Δ/Δ deletion mutants exhibit defective adhesion to TR146 cells.

Fig. S5 mbio.03304-25-s0005.pdf The *als3*Δ/Δ and *hgc1*Δ/Δ deletion mutants display invasion defects into TR146 cells.

Fig. S6 mbio.03304-25-s0006.tif Infection of epithelial cells with *ece1*Δ/Δ mutant strains does not activate MAPK signaling.

Fig. S7 mbio.03304-25-s0007.tif Additive gene deletion correlates to diminished immune activation and weight loss during OPC.

Supplemental material mbio.03304-25-s0008.docx Supplemental figure legends and tables.

ASM does not own the copyrights to Supplemental Material that may be linked to, or accessed through, an article. The authors have granted ASM a non-exclusive, world-wide license to publish the Supplemental Material files. Please contact the corresponding author directly for reuse.
