# Supplementary figures and images for: The combinatorial action of hyphal growth and candidalysin is critical for promoting Candida albicans oropharyngeal infection
Source: mBio. 2025 Nov 26;17(1):e03304-25. doi: 10.1128/mbio.03304-25 (PMC12802249; doi:10.1128/mbio.03304-25)

A

***ALS3***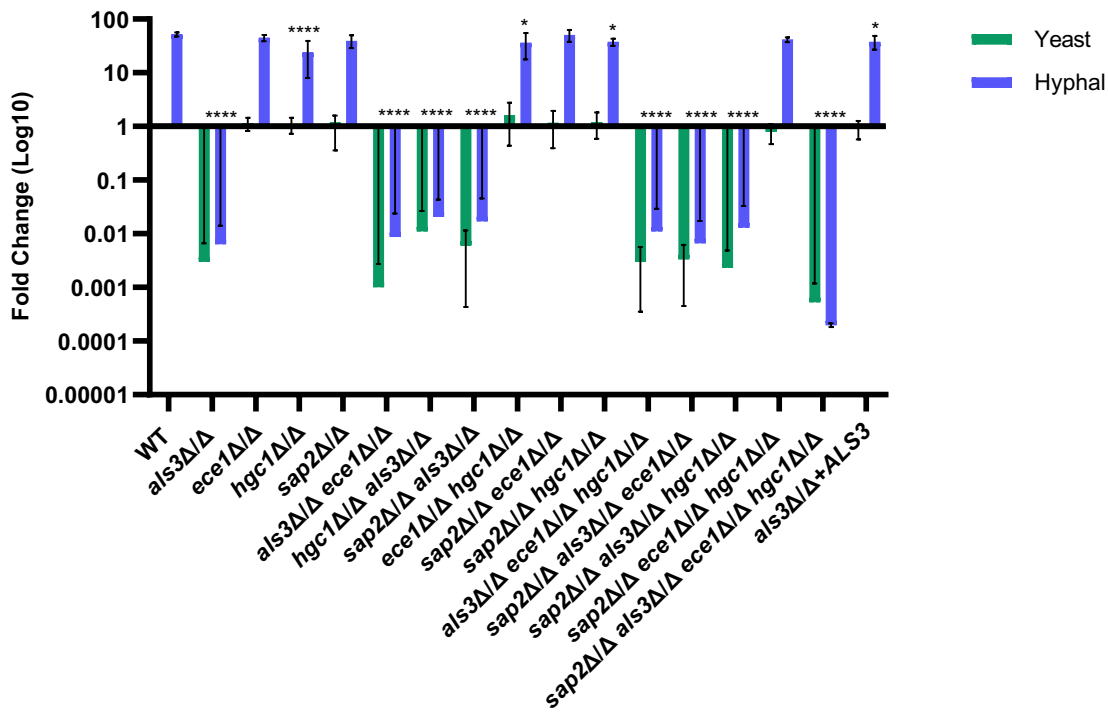

B

***ECE1***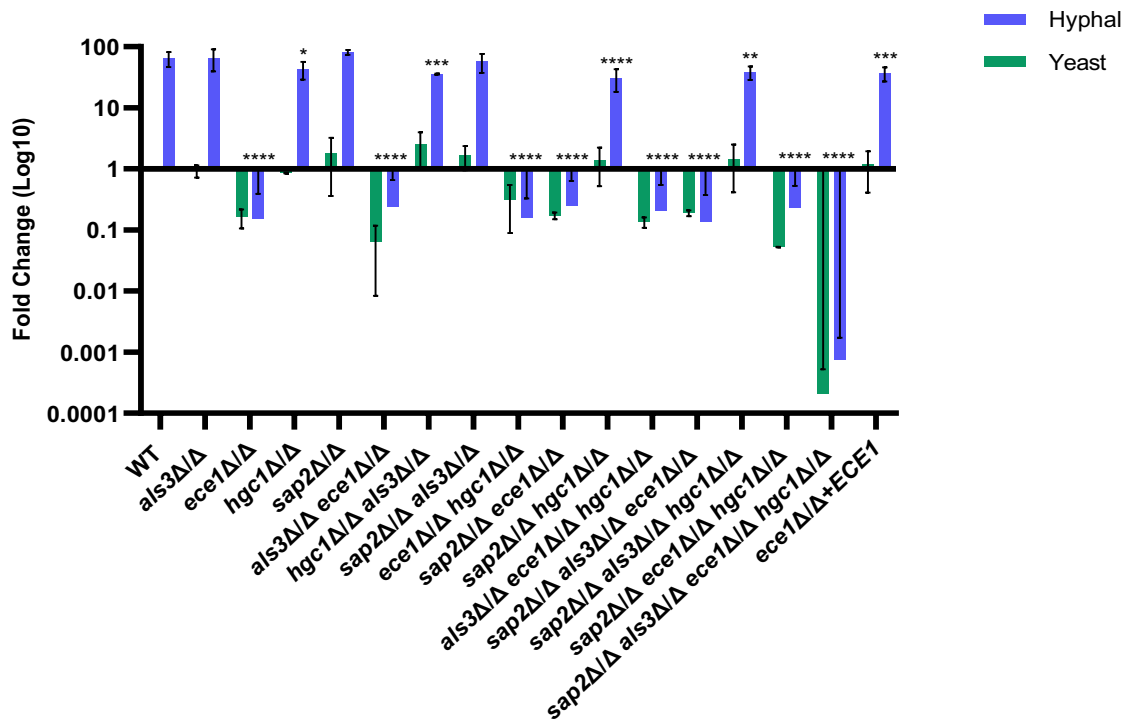

C

***HGC1***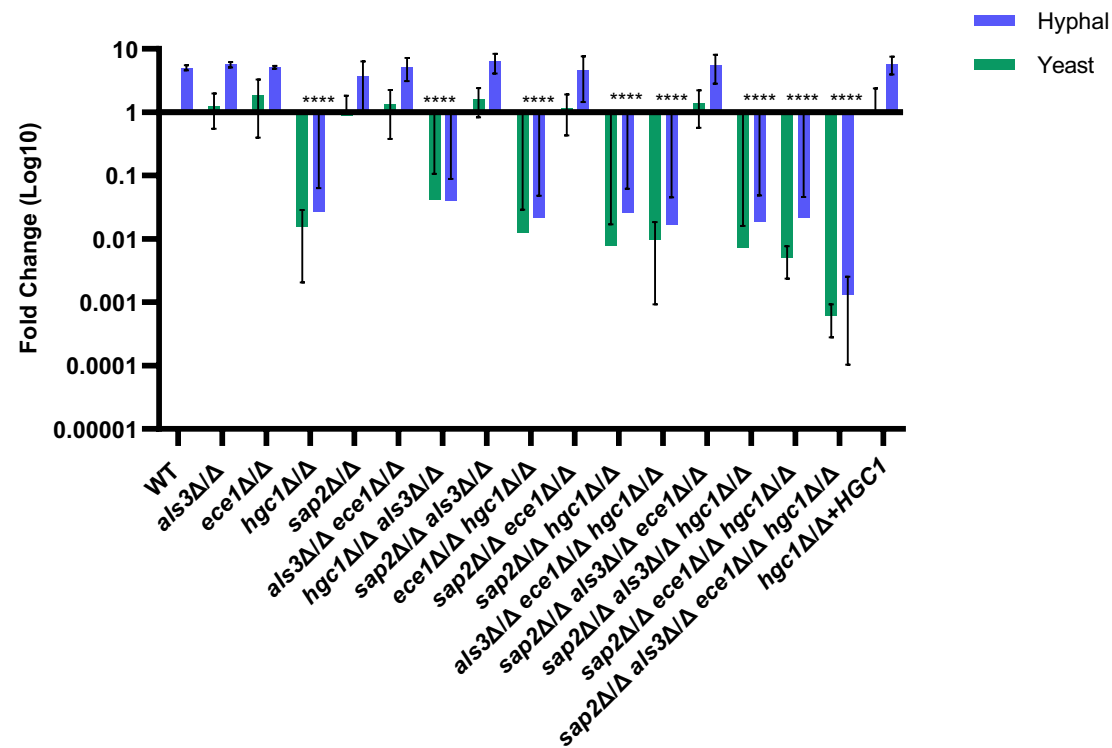

D i

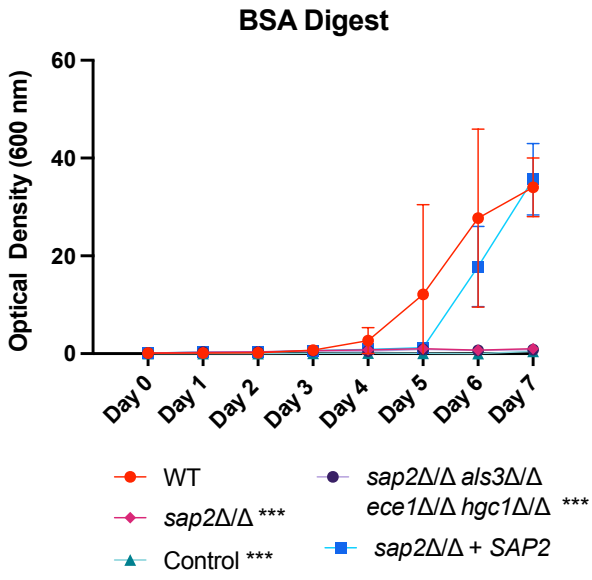

ii

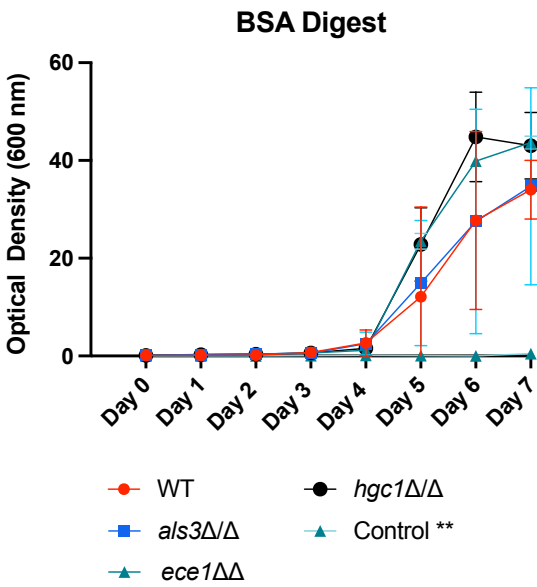

E

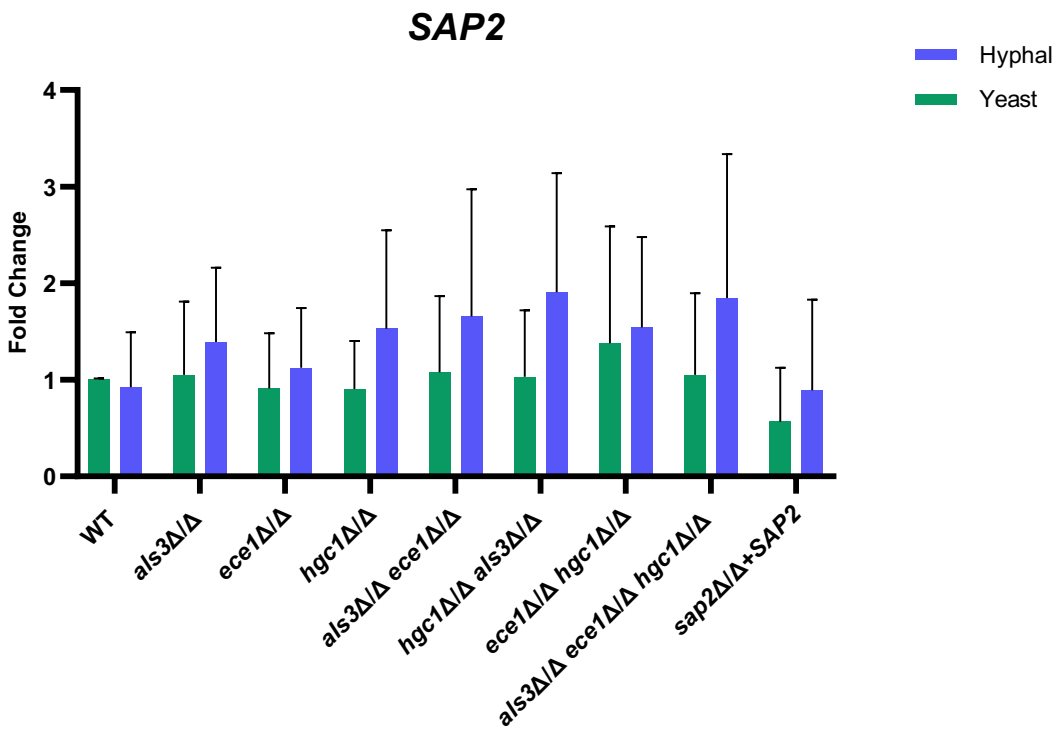

Supplement: Fig. S1 — Quantification of gene expression in C. albicans mutant strains constructed for this study. [file mbio.03304-25-s0001.pdf]

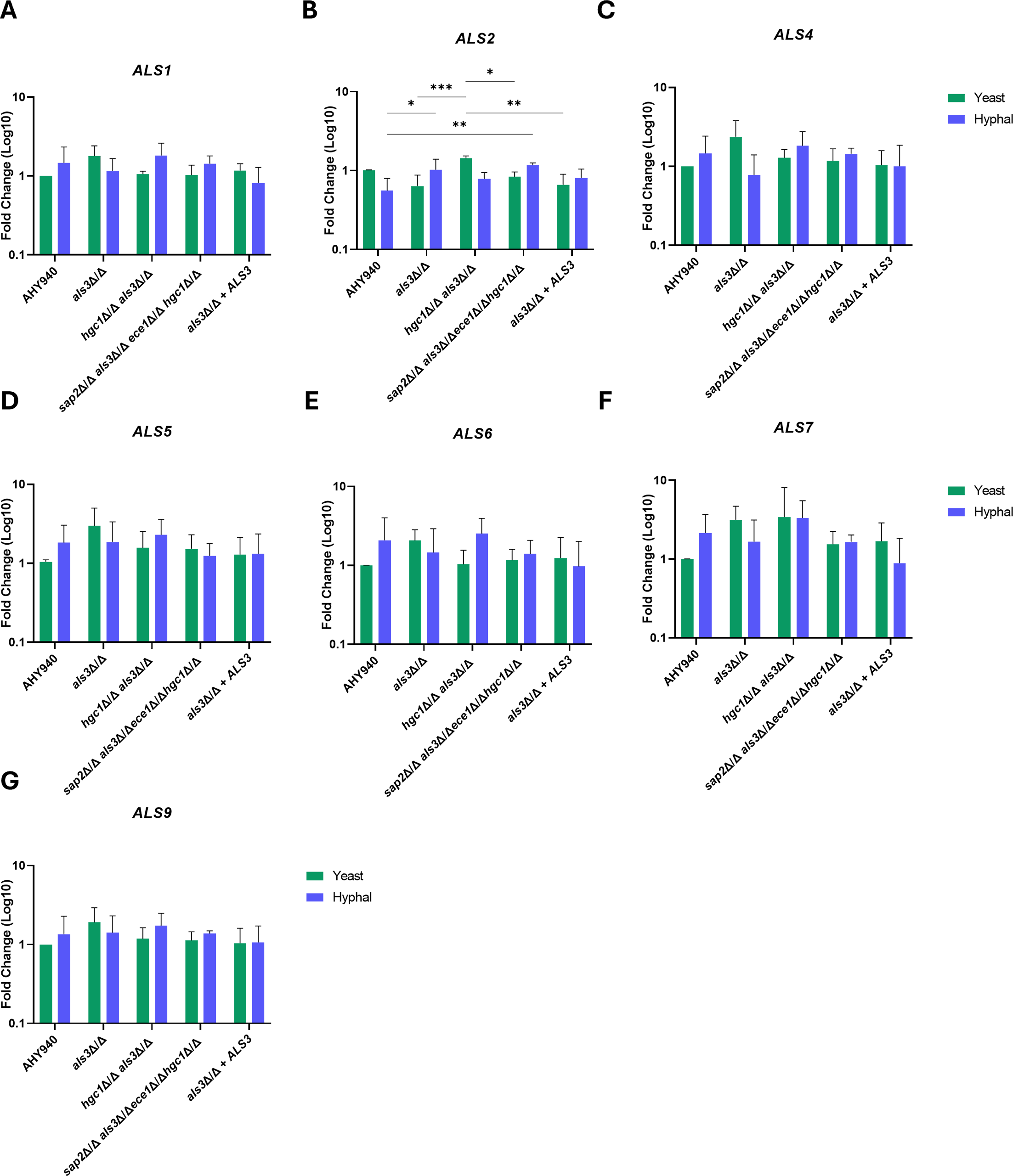

Supplement: Fig. S2 — Quantification of ALS family gene expression in selected C. albicans mutant strains. [file mbio.03304-25-s0002.tif]

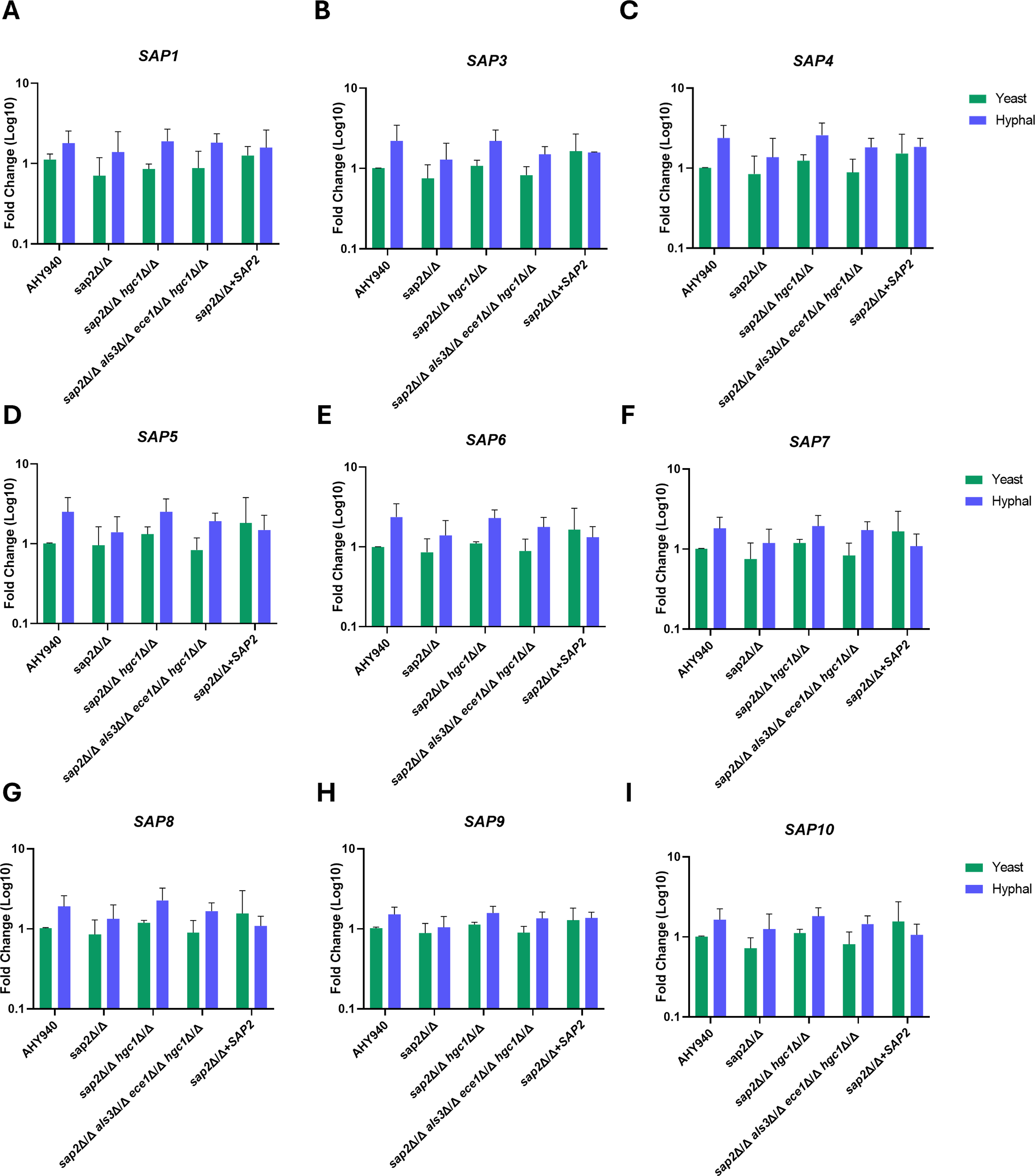

Supplement: Fig. S3 — Compensatory changes in SAP family expression are not observed in selected mutant strains. [file mbio.03304-25-s0003.tif]

**WT**

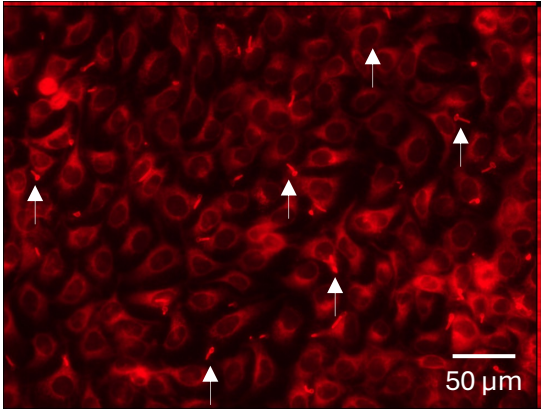

***a***

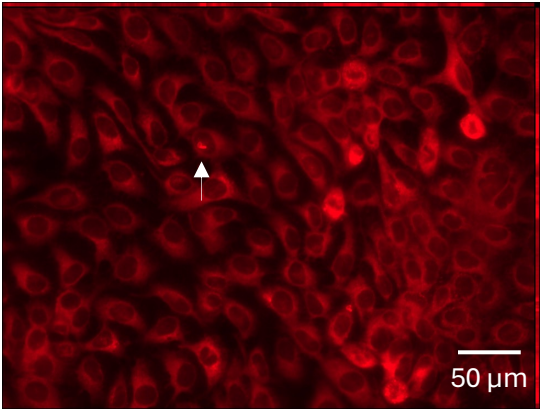

***e***

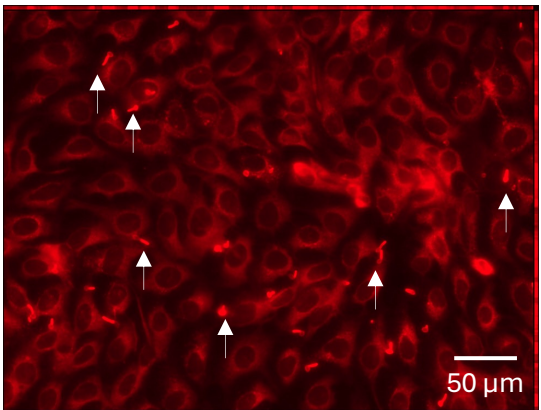

***h***

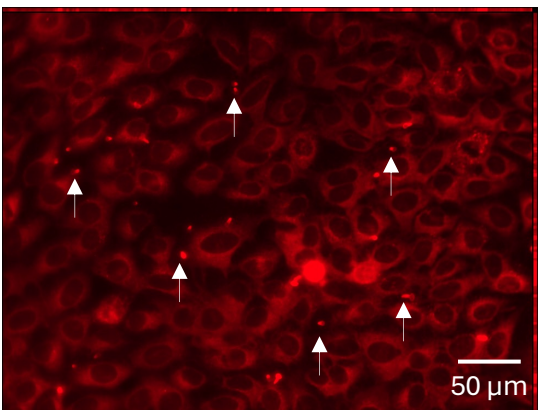

***s***

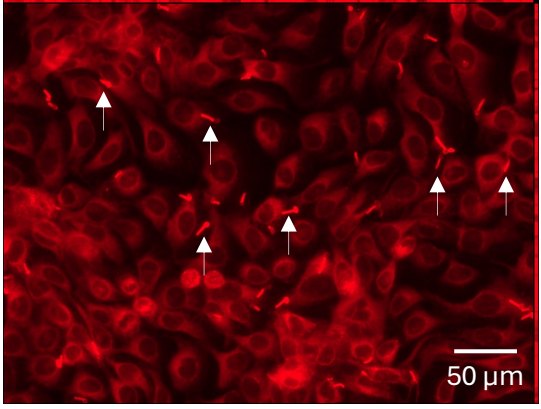

***ae***

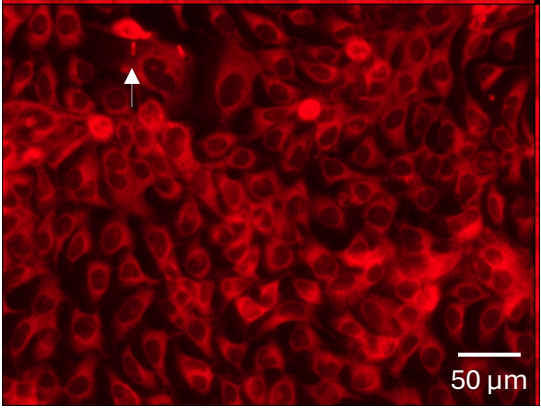

***ha***

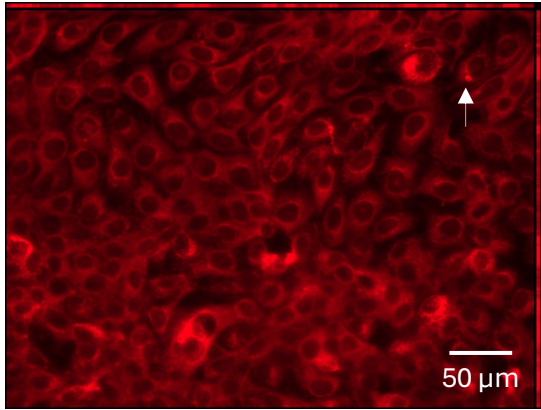

***sa***

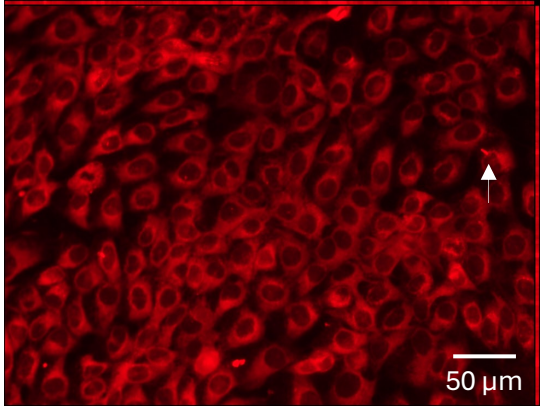

*eh*

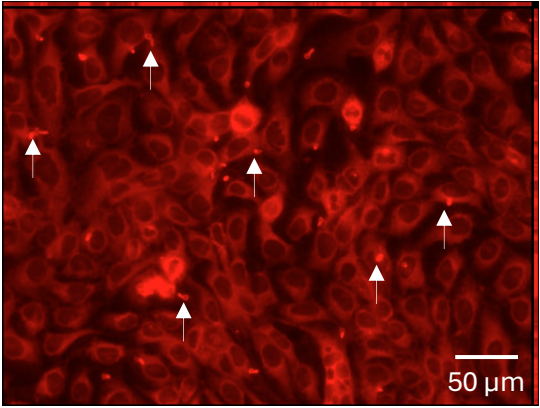

*se*

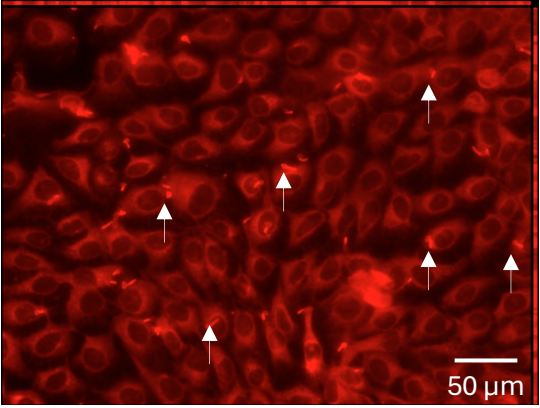

*sh*

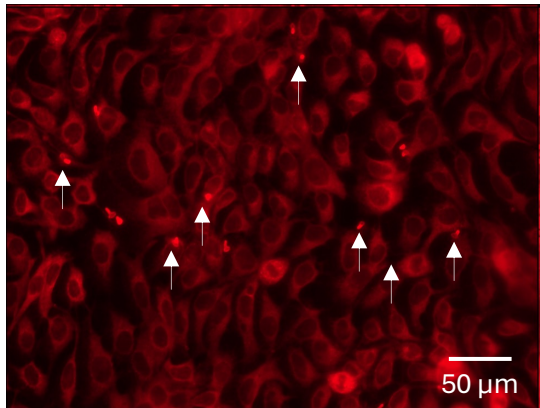

*aeh*

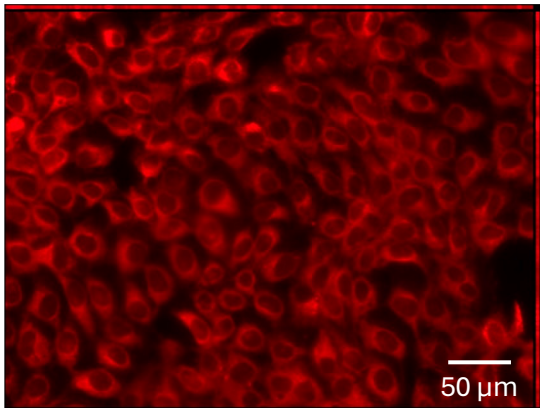

*sae*

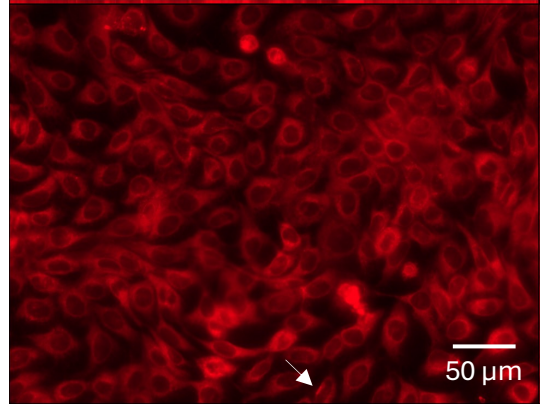

*sah*

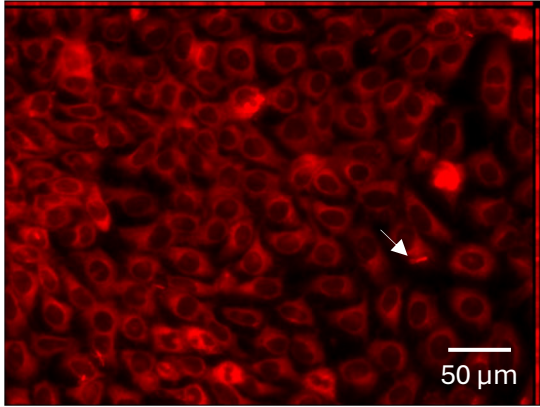

*seh*

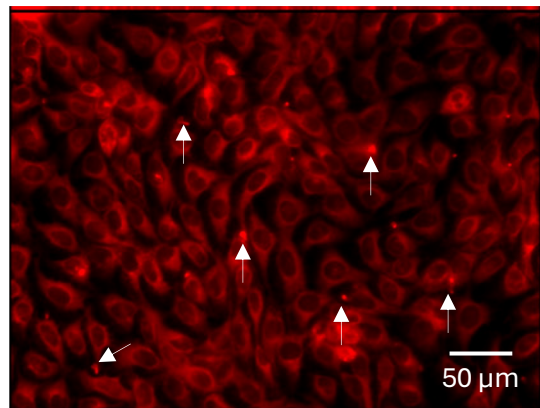

*saeh*

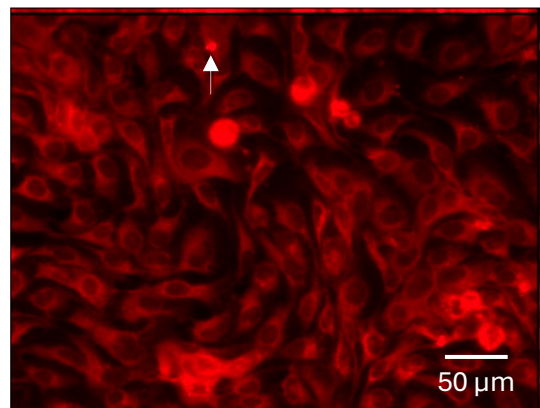

*a+A*

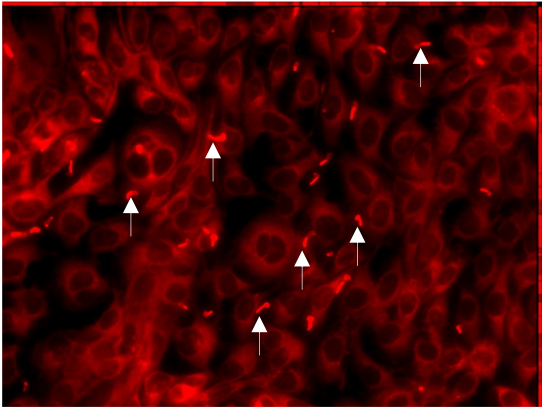

*e+E*

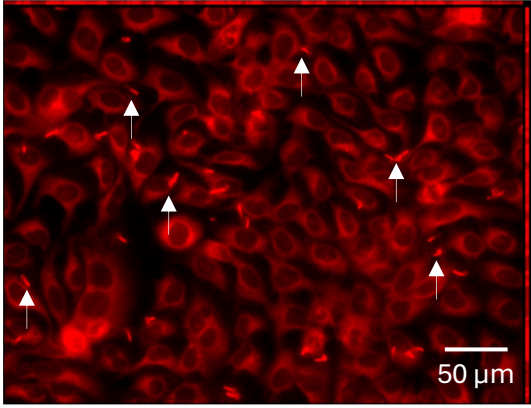

*h+H*

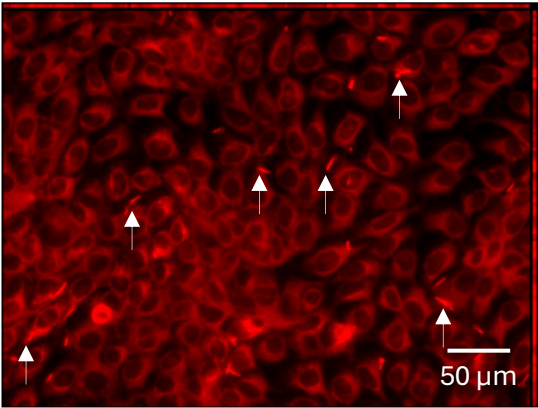

*s+S*

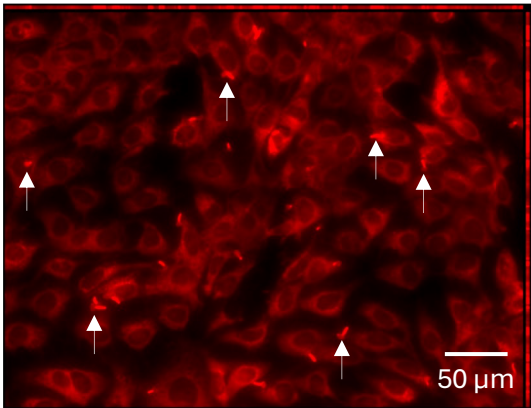

Supplement: Fig. S4 — C. albicans als3Δ/Δ deletion mutants exhibit defective adhesion to TR146 cells. [file mbio.03304-25-s0004.pdf]

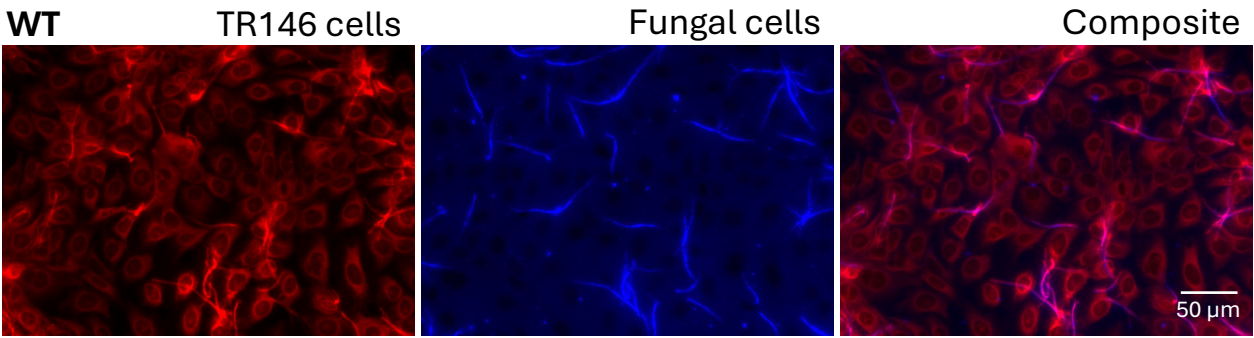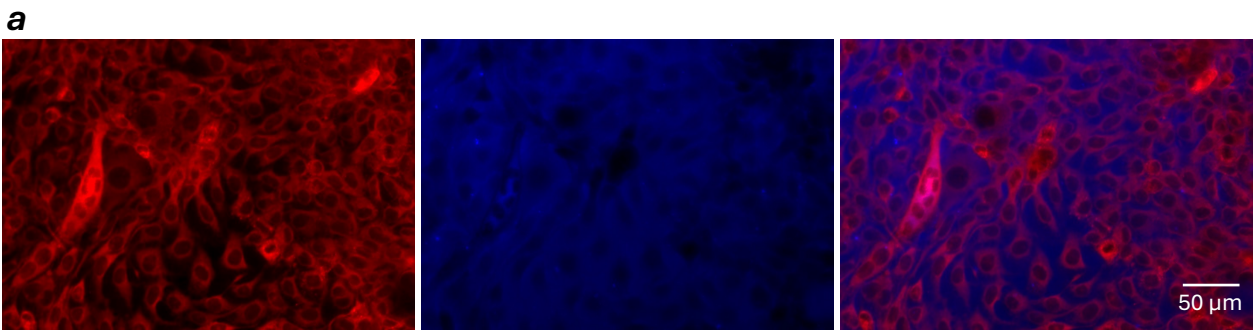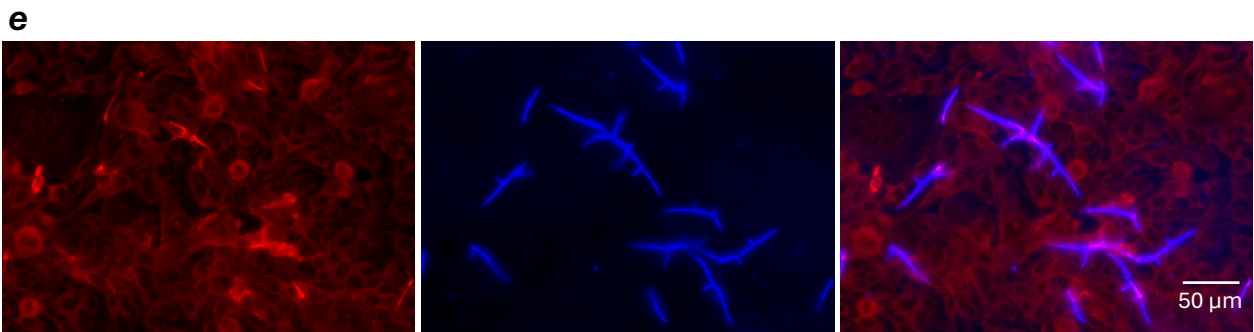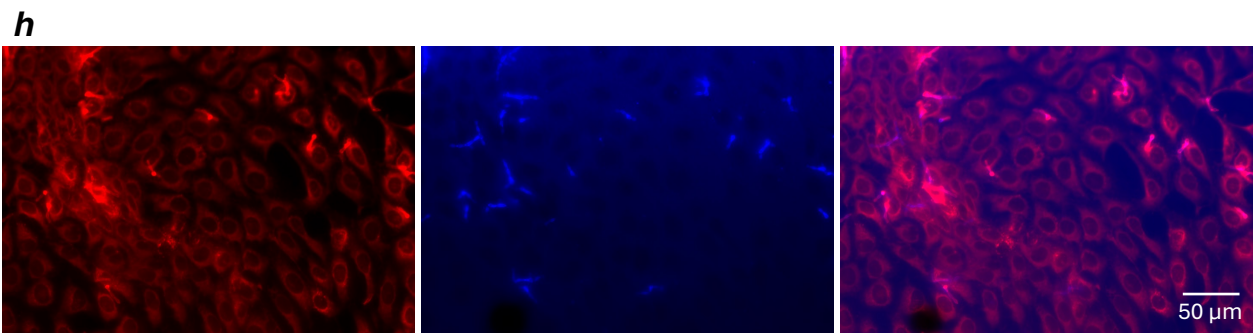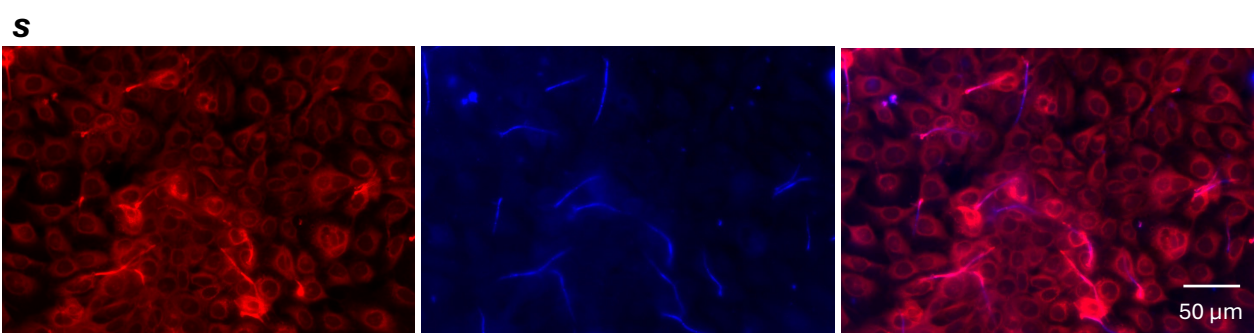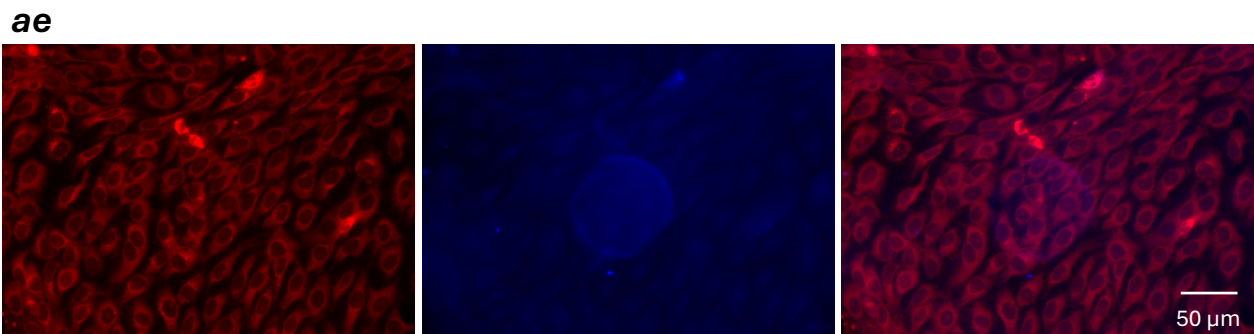

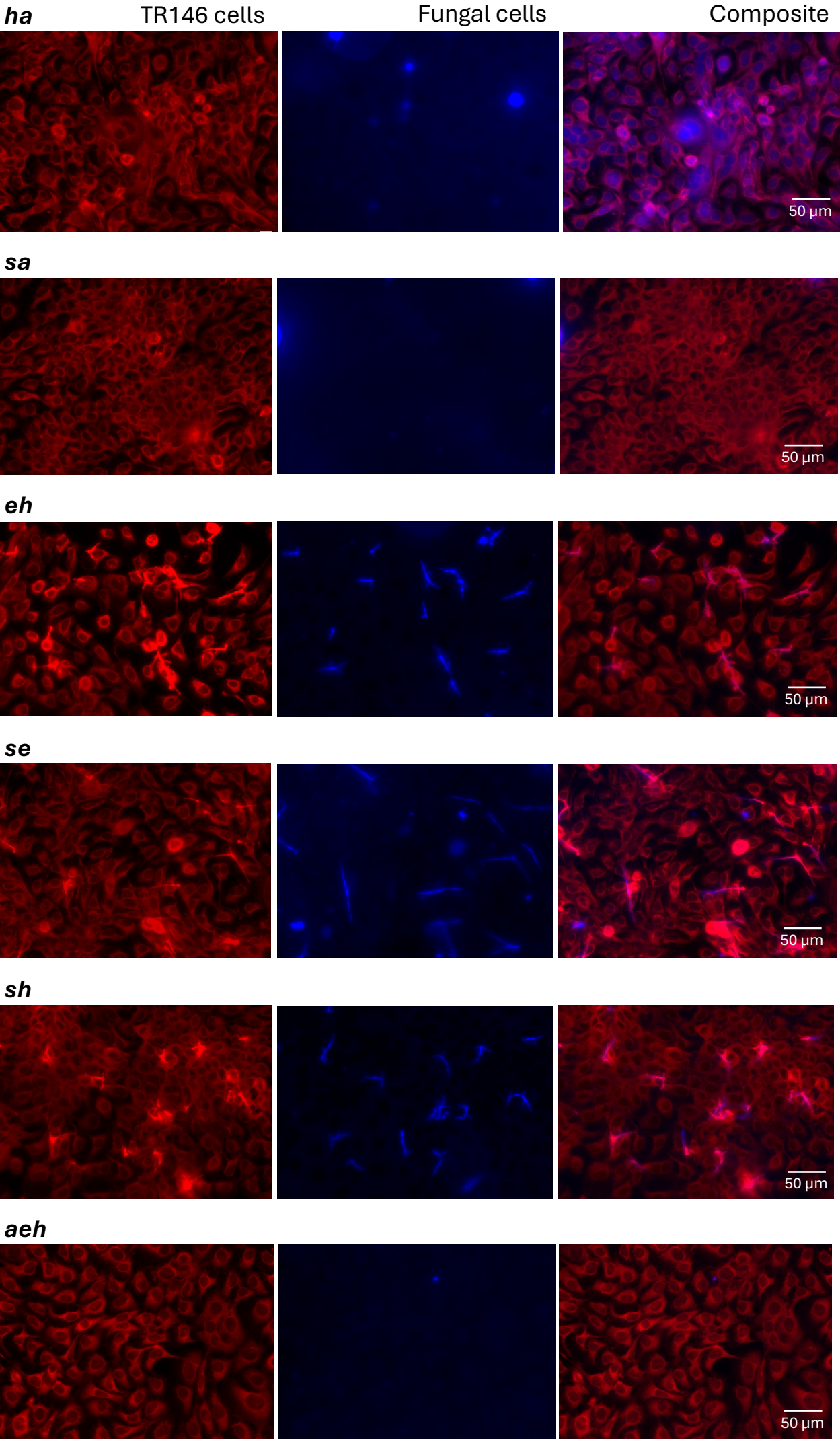

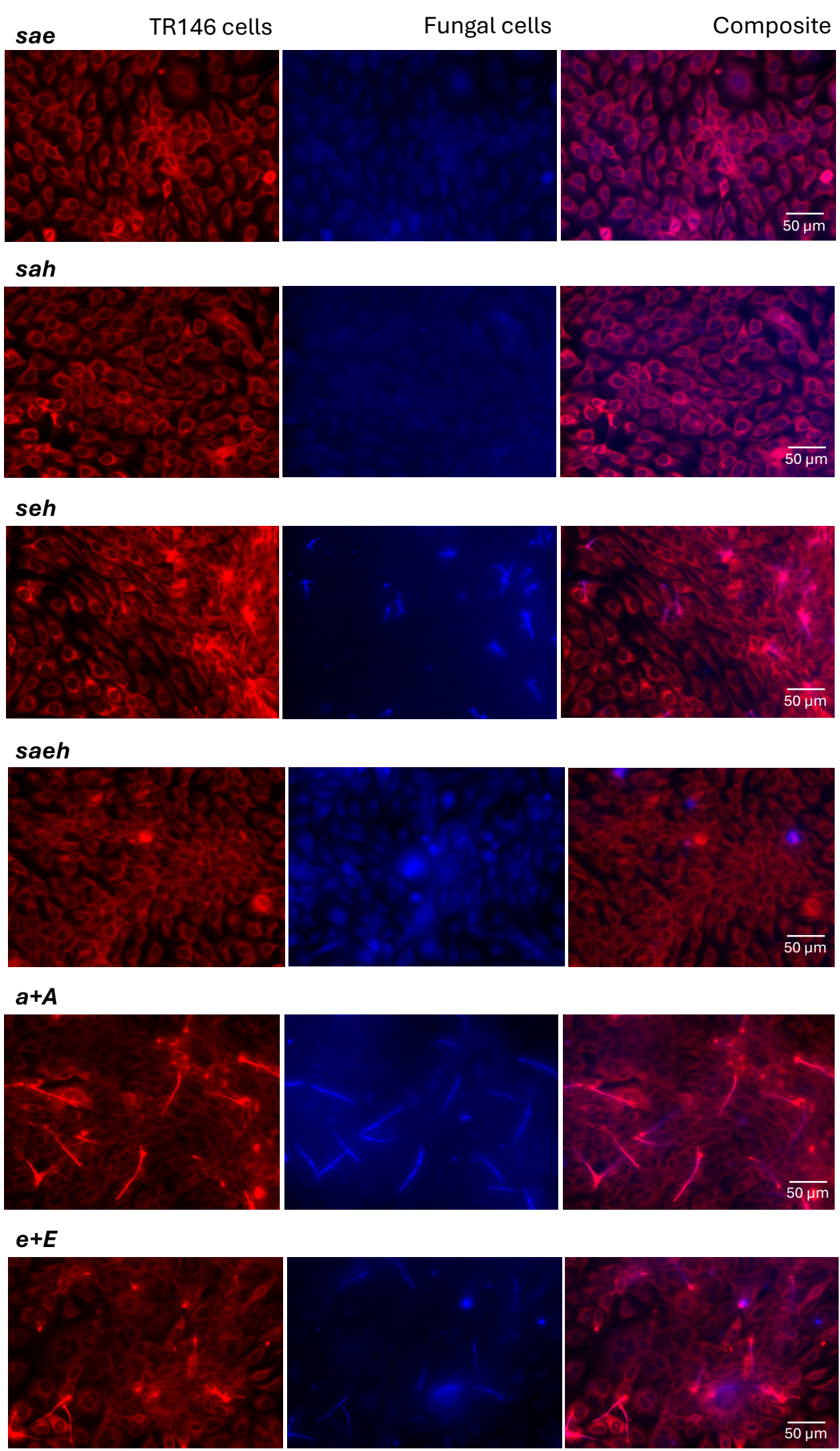

***h+H***                      TR146 cells                      Fungal cells                      Composite

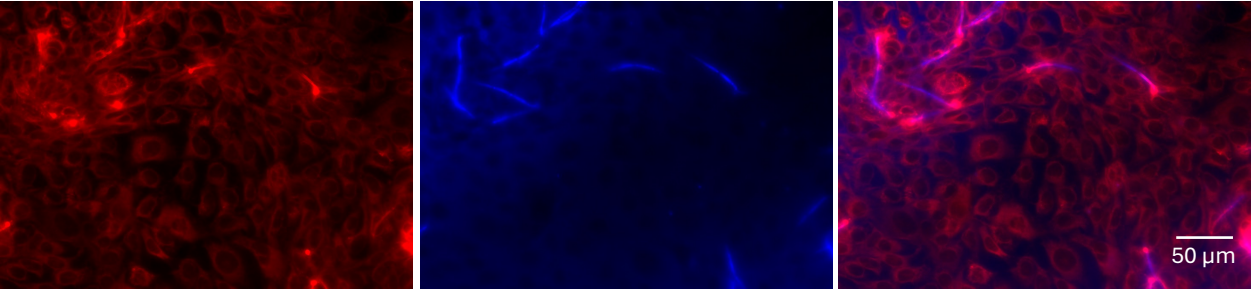

***s+S***

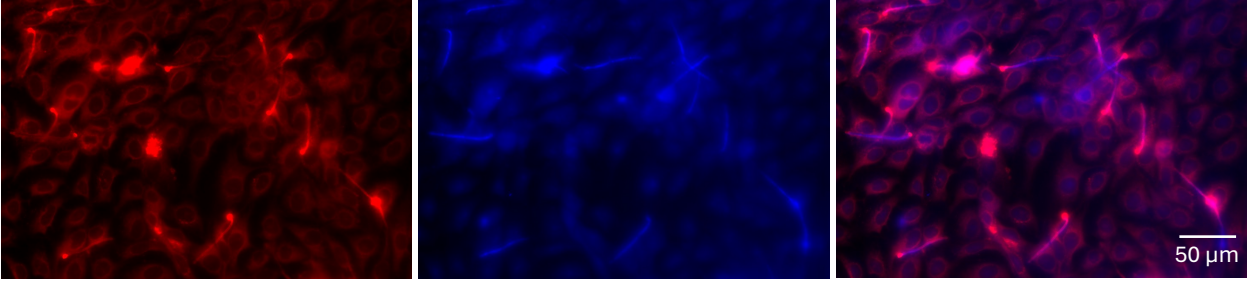

Supplement: Fig. S5 — The als3Δ/Δ and hgc1Δ/Δ deletion mutants display invasion defects into TR146 cells. [file mbio.03304-25-s0005.pdf]

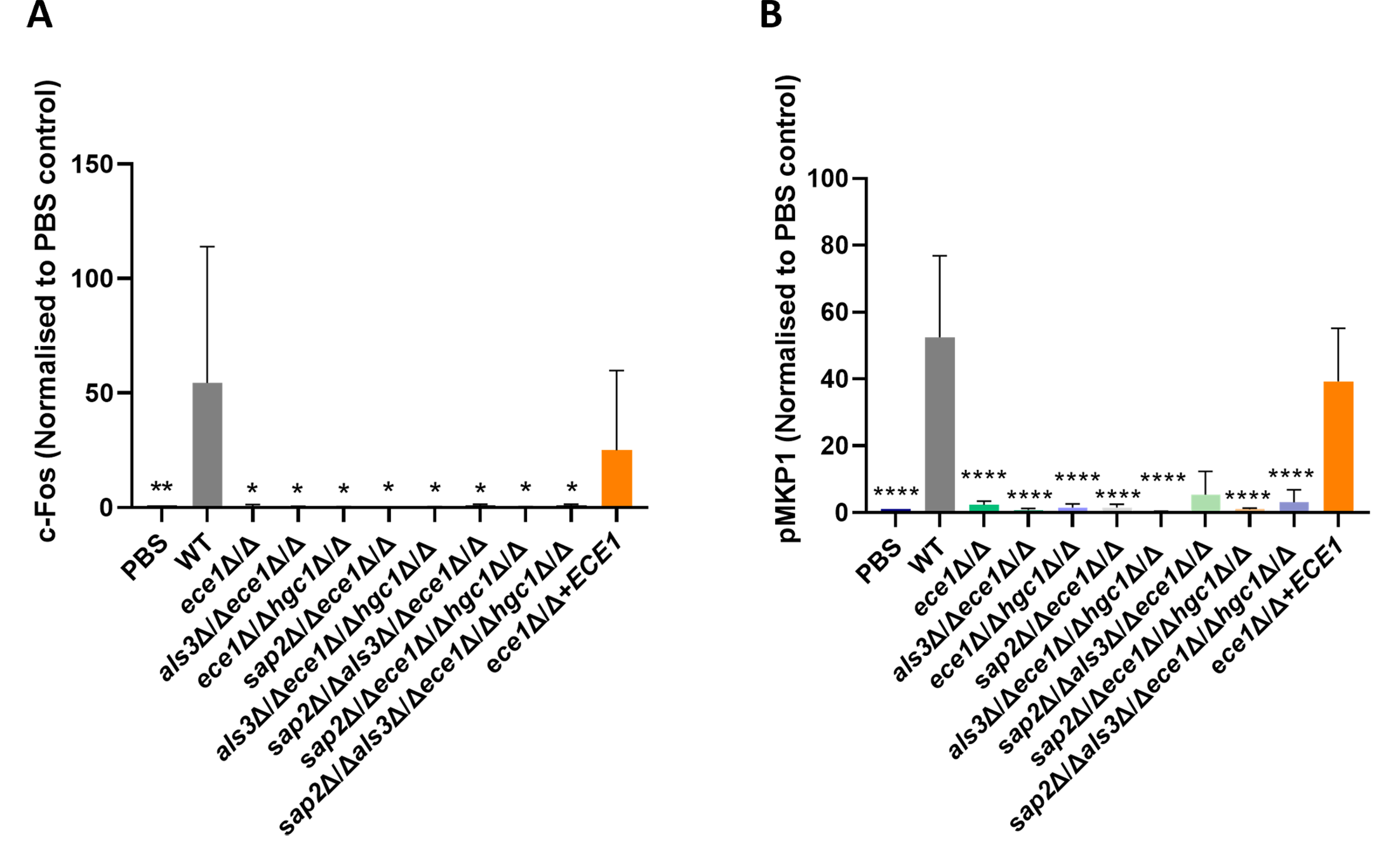

Supplement: Fig. S6 — Infection of epithelial cells with ece1Δ/Δ mutant strains does not activate MAPK signaling. [file mbio.03304-25-s0006.tif]

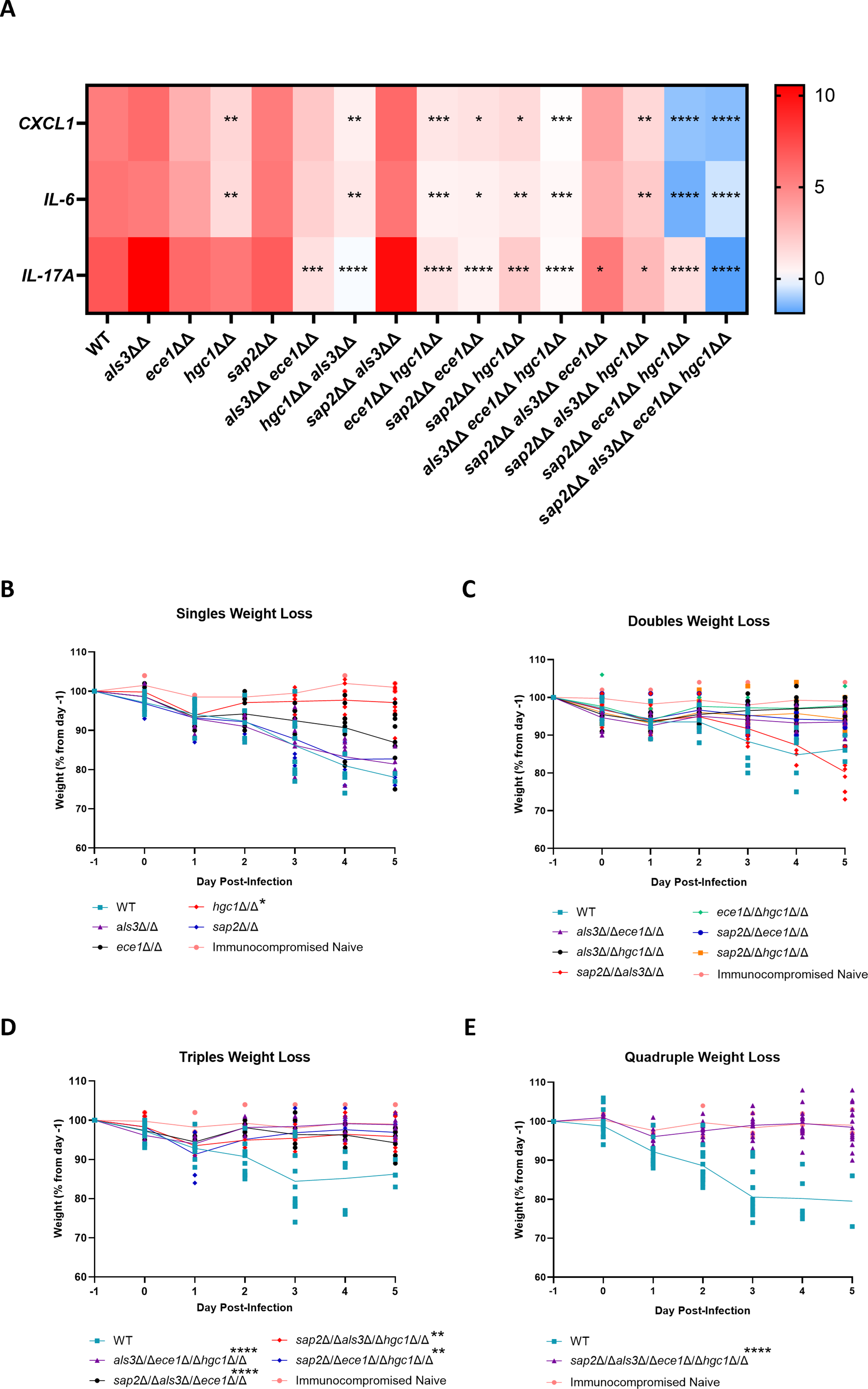

Supplement: Fig. S7 — Additive gene deletion correlates to diminished immune activation and weight loss during OPC. [file mbio.03304-25-s0007.tif]
