# Supplementary material for: The combinatorial action of hyphal growth and candidalysin is critical for promoting Candida albicans oropharyngeal infection
Source: mBio. 2025 Nov 26;17(1):e03304-25. doi: 10.1128/mbio.03304-25 (PMC12802249; doi:10.1128/mbio.03304-25)
Supplement: Supplemental material — Supplemental figure legends and tables. [file mbio.03304-25-s0008.docx]

# **Supplementary Figure Legends**

**Fig S1. Quantification of gene expression in *C. albicans* mutant strains created in this study. (A-C, E)** Strains were cultured in yeast and hypha-inducing conditions for 4 h and gene expression was quantified. Data is presented as log_10_-fold change (A-C), or fold change (E) normalised to yeast-WT and is the mean and standard deviation of three biological repeats. (A) *ALS3* (B) *ECE1* and (C) *HGC1* gene expression in AHY940 (WT) and all CRISPR strains. (D) Strains were grown in yeast carbon base (YCB) supplemented with 1% BSA at pH 4 for 7 days at 30˚ C, 200 RPM to induce expression of *SAP2*. Data is presented as an absorbance measurement taken every 24 h for 7 days for **i**) *sap2*Δ/Δ, *sap2*Δ/Δ *als3*Δ/Δ *ece1*Δ/Δ *hgc1*Δ/Δ and *sap2*Δ/Δ+*SAP2* and **ii**) WT, *als3*Δ/Δ, *ece1*Δ/Δ, *hgc1*Δ/Δ strains. (E) *SAP2* gene expression in AHY940 (WT) and all non-*sap2*∆/∆ CRISPR strains. All data is the mean of three biological replicates. Statistical significance was calculated using two-way ANOVA using a Dunnett’s comparison test compared to AHY940 (WT). ****, P > 0.0001; ***, P > 0.001; **, P > 0.01; *, P > 0.05.

**Fig S2. Quantification of *ALS* family gene expression in selected *C. albicans* mutant strains.** Strains were cultured in yeast and hypha-inducing conditions for 2.5 h and gene expression was quantified. Data is presented as log_10_-fold change normalised to yeast-WT and is the mean and standard deviation of three biological repeats. Expression of (A) *ALS1*, (B) *ALS2*, (C) *ALS4*, (D) *ALS5*, (E) *ALS6*, (F) *ALS7*, and (G) *ALS9* in AHY940 (WT), *als3*∆/∆, *hgc1*∆/∆ *als3*∆/∆, *sap2*∆/∆ *als3*∆/∆ *ece1*∆/∆ *hgc1*∆/∆ and *als3*∆/∆+*ALS3* strains. Statistical significance was calculated using two-way ANOVA using a Dunnett’s comparison test compared to AHY940 (WT) Yeast and AHY940 Hyphal. ***, P > 0.001; **, P > 0.01; *, P > 0.05.

**Fig S3. Compensatory changes in *SAP* family expression are not observed in selected mutant strains.**  Strains were cultured in yeast and hypha-inducing conditions for 4 h and gene expression was quantified. Data is presented as log_10_-fold change normalised to yeast-WT and is the mean and standard deviation of three biological repeats. RT-qPCR data for (A) *SAP1*, (B) *SAP3*, (C) *SAP4*, (D) *SAP5*, (E) *SAP6*, (F) *SAP7*, (G) *SAP8*, (H) *SAP9*, (I) *SAP10* gene expression in AHY940 (WT), *sap2*∆/∆, *sap2*∆/∆ *hgc1*∆/∆, *sap2*∆/∆ *als3*∆/∆ *ece1*∆/∆ *hgc1*∆/∆ and *sap2*∆/∆+*SAP2* strains*.* Statistical significance was calculated using two-way ANOVA using a Dunnett’s comparison test compared to AHY940 (WT) Yeast and AHY940 Hyphal.

**Fig S4.** ***C. albicans* *als3*Δ/Δ deletion mutants exhibit defective adhesion to TR146 cells.** TR146 cells were infected with strains (MOI 1) for 1 h before staining with Concanavalin A. Numbers of fungal cells adhered were counted and data is presented as representative microscopy images. Numerical data is presented in **Fig 1D**. TR146 cells were infected with AHY940 (**WT**), *als3*Δ/Δ (***a***)*, ece1*Δ/Δ (***e***)**,** *hgc1*Δ/Δ (***h***)**,** *sap2*Δ/Δ (***s***)**,** *als3*Δ/Δ *ece1*Δ/Δ (***ae***)**,** *hgc1*Δ/Δ *als3*Δ/Δ (***ha***)**,** *sap2*Δ/Δ *als3*Δ/Δ (***sa***)**,** *ece1*Δ/Δ *hgc1*Δ/Δ (***eh***)*, sap2*Δ/Δ *ece1*Δ/Δ (***se***)**,** *sap2*Δ/Δ *hgc1*Δ/Δ (***sh***), *als3*Δ/Δ *ece1*Δ/Δ *hgc1*Δ/Δ (***aeh***), *sap2*Δ/Δ *als3*Δ/Δ *ece1*Δ/Δ (***sae***), *sap2*Δ/Δ *als3*Δ/Δ *hgc1*Δ/Δ (***sah***), *sap2*Δ/Δ *ece1*Δ/Δ *hgc1*Δ/Δ (***seh***), *sap2*Δ/Δ *als3*Δ/Δ *ece1*Δ/Δ *hgc1*Δ/Δ (***saeh***), *als3*Δ/Δ+*ALS3* (***a*+*A***), *ece1*Δ/Δ+*ECE1* (***e*+*E***), *hgc1*Δ/Δ+*HGC1* (***h*+*H***),and *sap2*Δ/Δ+*SAP2* (***s*+*S***). White arrows point at selected fungal cells. Data representative of three biological repeats. Scale bar represents 50 µm.

**S5 Fig S5.** **The *als3*Δ/Δ and *hgc1*Δ/Δ deletion mutants display invasion defects into TR146 cells.** TR146 cells were infected with strains (MOI 1) for 4 h before staining with Concanavalin and calcofluor white. Data is presented as TR146 cells, fungal cells and a composite image. Numerical data is presented in **Fig 1F.** TR146 cells were infected with AHY940 (**WT**), *als3*Δ/Δ (***a***)*, ece1*Δ/Δ (***e***)**,** *hgc1*Δ/Δ (***h***)**,** *sap2*Δ/Δ (***s***)**,** *als3*Δ/Δ *ece1*Δ/Δ (***ae***)**,** *hgc1*Δ/Δ *als3*Δ/Δ (***ha***)**,** *sap2*Δ/Δ *als3*Δ/Δ (***sa***)**,** *ece1*Δ/Δ *hgc1*Δ/Δ (***eh***)*, sap2*Δ/Δ *ece1*Δ/Δ (***se***)**,** *sap2*Δ/Δ *hgc1*Δ/Δ (***sh***), *als3*Δ/Δ *ece1*Δ/Δ *hgc1*Δ/Δ (***aeh***), *sap2*Δ/Δ *als3*Δ/Δ *ece1*Δ/Δ (***sae***), *sap2*Δ/Δ *als3*Δ/Δ *hgc1*Δ/Δ (***sah***), *sap2*Δ/Δ *ece1*Δ/Δ *hgc1*Δ/Δ (***seh***), *sap2*Δ/Δ *als3*Δ/Δ *ece1*Δ/Δ *hgc1*Δ/Δ (***saeh***), *als3*Δ/Δ+*ALS3* (***a*+*A***), *ece1*Δ/Δ+*ECE1* (***e*+*E***), *hgc1*Δ/Δ+*HGC1* (***h*+*H***) and *sap2*Δ/Δ+*SAP2* (***s*+*S***). Data are representative of three biological repeats. Scale bar represents 50 μm.

**Fig S6.** **Infection of epithelial cells with *ece1*Δ/Δ mutant strains does not activate MAPK signalling.** TR146 cells were infected with PBS, AHY940 (WT), and all *ece1*∆/∆ mutant strains for 2 h (MOI 10). Samples containing 10 μg of total protein were electrophoresed on SDS-PAGE 4-12% gradient gels and analysed by western blot. Densitometry analysis was performed for (A) c-Fos expression and (B) MKP1 phosphorylation. All values were actin-normalised and are presented relative to the PBS-treated control, which was arbitrarily assigned a value of 1. Data is the mean of three biological replicates Statistical significance was calculated using one-way ANOVA using a Dunnett’s comparison test fixed to WT (AHY940). ****, P > 0.0001; **, P > 0.01; *, P > 0.05.

**Fig S7. Additive gene deletion correlates to diminished immune activation and weight loss during OPC.** Immunocompromised mice were infected for 75 min using a sublingual swab soaked in a 1x10^7^ cells/mL solution of *C. albicans* and a total of three doses of cortisone acetate were administered on D-1, D1 and D3 post-infection to maintain immunosuppression. (A) Heatmap depicting RT-qPCR analysis performed on tongue tissue for genes encoding for immune components. Data is presented as log_2_ fold change of *CXCL1*, *IL-6* and *IL-17A* expression relative to the naïve control. Data is pooled from day of culling (if ≤80% original weight) or D5. (B-E) Weights were monitored daily for 5 days post-infection. Data has been separated into infection with (B) Single, (C) Double, (D) Triple and (E) Quadruple deletion mutants compared to WT (AHY940). Data is representative of two biological replicates (n=8-10 mice) and (B-E) is presented as the mean (singular line) with individual values plotted as dots. For all datasets, statistical significance was calculated using an (A) two-way ANOVA or (B-E) one-way ANOVA using a Dunnett’s comparison test fixed to AHY940 (WT). For weight loss analyses, statistics were performed at D2 as this was before any weight-loss associated culling of mice. ****, P < 0.0001; ***, P < 0.001; **, P < 0.01; *, P < 0.05.

## **Supplementary Tables**

**Table S1**. **Expression of cytokines/chemokines following a murine OPC infection with mutant strains.** Datasets are relative to a naïve control and are presented as the mean value, obtained from mice at D3-5 post-infection. Statistical significance was calculated using a two-way ANOVA with a Dunnett’s comparison test fixed to AHY940 (WT). ****, P < 0.0001; ***, P < 0.001; **, P < 0.01; *, P < 0.05.

|  | ***CXCL1*** | ***IL-6*** | ***IL17-A*** |
| --- | --- | --- | --- |
| WT (AHY940) | 70.45 | 113.08 | 1479.55 |
| *als3*∆/∆ | 58.72 | 51.99 | 1691.78 |
| *ece1*∆/∆ | 13.78 | 14.52 | 759.11 |
| *hgc1*∆/∆ | 5.18 ** | 3.32 ** | 1081.60 |
| *sap2*∆/∆ | 69.39 | 98.21 | 2035.23 |
| *als3*∆/∆ *ece1*∆/∆ | 12.40 | 10.39 | 234.88 *** |
| *hgc1*∆/∆ *als3*∆/∆ | 3.07 ** | 3.26 ** | 2.66 **** |
| *sap2*∆/∆ *als3*∆/∆ | 79.93 | 103.16 | 2878.91 |
| *ece1*∆/∆ *hgc1*∆/∆ | 1.81 *** | 1.52 *** | 7.28 **** |
| *sap2*∆/∆ *ece1*∆/∆ | 8.83 * | 12.18 * | 89.26 **** |
| *sap2*∆/∆ *hgc1*∆/∆ | 4.32 * | 3.38 ** | 9.41 *** |
| *als3*∆/∆ *ece1*∆/∆ *hgc1*∆/∆ | 0.98 *** | 1.26 *** | 0.79 **** |
| *sap2*∆/∆ *als3*∆/∆ *ece1*∆/∆ | 18.22 | 17.50 | 336.00 * |
| *sap2*∆/∆ *als3*∆/∆ *hgc1*∆/∆ | 3.76 ** | 4.98 ** | 159.58 * |
| *sap2*∆/∆ *ece1*∆/∆ *hgc1*∆/∆ | 0.51 **** | 0.92 **** | 189.89 **** |
| *sap2*∆/∆ *als3*∆/∆ *ece1*∆/∆ *hgc1*∆/∆ | 0.48 **** | 1.31 **** | 171.54 **** |

**Table S2**. **Expression of cytokines/chemokines following a two-day murine OPC infection with mutant strains.** Datasets are relative to a naïve control and are presented as the mean value, obtained from mice at day 2 post-infection. Statistical significance was calculated using a two-way ANOVA with a Dunnett’s comparison test fixed to AHY940 (WT). ****, P < 0.0001; ***, P < 0.001; **, P < 0.01; *, P < 0.05.

|  | ***CXCL1*** | ***IL-6*** | ***IL17-A*** | ***S100A9*** | ***TNF*** |
| --- | --- | --- | --- | --- | --- |
| WT (AHY940) | 85.24 | 78.51 | 584.16 | 1099.66 | 49.08 |
| *als3*∆/∆ | 44.53 | 39.94 | 238.16 | 486.53 | 37.42 |
| *ece1*∆/∆ | 13.48 ** | 51.25 | 5.07**** | 37.02 **** | 9.09 * |
| *hgc1*∆/∆ | 4.46 **** | 5.62 *** | 31.57 **** | 18.64 **** | 3.28 **** |
| *sap2*∆/∆ | 85.83 | 92.14 | 601.15 | 588.14 | 45.82 |
| *sap2*∆/∆ *als3*∆/∆ | 69.42 | 94.16 | 641.09 | 778.43 * | 39.29 |
| *ece1*∆/∆ *hgc1*∆/∆ | 1.23 **** | 2.19 **** | 0.85 **** | 0.94 **** | 0.93 **** |
| *als3*∆/∆ *ece1*∆/∆ *hgc1*∆/∆ | 1.86 **** | 4.04 **** | 1.65 **** | 5.54 **** | 1.37 **** |
| *sap2*∆/∆ *ece1*∆/∆ *hgc1*∆/∆ | 0.85 **** | 1.02 **** | 0.79 **** | 0.86 **** | 0.93 **** |
| *sap2*∆/∆ *als3*∆/∆ *ece1*∆/∆ *hgc1*∆/∆ | 1.44 **** | 2.62 **** | 1.17 **** | 2.22 **** | 0.98 **** |

**Table S3. Oligonucleotides used in this study.**

| **Oligo Name** | **FWD/REV** | **FUNCTION** | **DESCRIPTION** | **SEQUENCE (5' to 3')** |
| --- | --- | --- | --- | --- |
| AHO1096 | FWD | PCR | Fragment A | GACGGCACGGCCACGCGTTTAAACCGCC |
| AH01098 | REV | PCR | Fragment A | CAAATTAAAAATAGTTTACGCAAG |
| AHO1097 | REV | PCR | Fragment B | CCCGCCAGGCGCTGGGGTTTAAACACCG |
| AHO1237 | FWD | PCR | Fragment C | AGGTGATGCTGAAGCTATTGAAG |
| AHO1238 | REV | PCR | Fragment C | TGTATTTTGTTTTAAAATTTTAGTGACTGTTTC |
| OPO1 | FWD | gRNA | *ALS3* ORF gRNA | CGTAAACTATTTTTAATTTGACGTATAATTATAAGGGACCGTTTTAGAGCTAGAAATAGC |
| OPO2 | FWD | dDNA | *ALS3* dDNA (deletion) | ATTTCATTTTATTATAATTGTATAAACAACTACCAACTGCTAATATTAGGGGGATGATACCATCATAGTCGCCTTTTTAGATTTTTGTTATTTGTTTGTT |
| OPO3 | REV | dDNA | *ALS3* dDNA (deletion) | AACAAACAAATAACAAAAATCTAAAAAGGCGACTATGATGGTATCATCCCCCTAATATTAGCAGTTGGTAGTTGTTTATACAATTATAATAAAATGAAAT |
| OPO4 | FWD | gRNA | *ALS3* gRNA (mADD-TAG) | CGTAAACTATTTTTAATTTGACTACCAACTGCTAATATTAGTTTTAGAGCTAGAAATAGC |
| OPO5 | FWD | cPCR/ADD-BACK dDNA PCR/Sequencing | *ALS3* flanking primer (500 bp u/s of ORF) | CCTTATGGAGCTATTGGCAAC |
| OPO6 | REV | cPCR/ADD-BACK dDNA PCR | *ALS3* reverse flanking primer (500 bp d/s of ORF) | TTCTAACACCTCAACTTAGTAGCC |
| OPO7 | FWD | cPCR | *ALS3* ORF specific primer | ACTTGGTCTAATGCTGCTACG |
| OPO8 | REV | cPCR | *ALS3* ORF specific primer | GTTGAAGTTGCAGATGGAGC |
| OPO94 | FWD | cPCR/Sequencing | *ALS3* flanking | TGCACGTTCATACTTCCAAAA |
| OPO95 | REV | cPCR | *ALS3* flanking | CAATTCATAGTCGCAGGCTCT |
| OPO102 | FWD | cPCR | *ALS3* flanking | CGTGCATAAGAAAGTTTTGCTATGC |
| OPO103 | RED | cPCR | *ALS3* flanking | CTGAACAATTCATAGTCGCAGGC |
| OPO17 | REV | gRNA | *ECE1* ORF gRNA | CGTAAACTATTTTTAATTTGTGTTGAATTCTGGAGCATGGGTTTTAGAGCTAGAAATAGC |
| OPO18 | FWD | dDNA | *ECE1* dDNA (deletion) | ACAAACAACTTTCCTTTATTTTACTACCAACTATTTTCCATTCGTTAAAGGATGCTCAGCAGATAAAAATTTGTTTTCCACAAGCTTAATCTTTTATTCC |
| OPO19 | REV | dDNA | *ECE1* dDNA (deletion) | GGAATAAAAGATTAAGCTTGTGGAAAACAAATTTTTATCTGCTGAGCATCCTTTAACGAATGGAAAATAGTTGGTAGTAAAATAAAGGAAAGTTGTTTGT |
| OPO20 | FWD | gRNA | *ECE1* gRNA (mADD-TAG) | CGTAAACTATTTTTAATTTGAACTATTTTCCATTCGTTAAGTTTTAGAGCTAGAAATAGC |
| OPO23 | FWD | cPCR | *ECE1* ORF specific primer | CATCATCCACCATGCTCC |
| OPO24 | REV | cPCR | *ECE1* ORF specific primer | AACATCTGGAACGCCATC |
| OPO41 (2) | REV | gRNA (Thad) | *ECE1* gRNA (Thad) | CGTAAACTATTTTTAATTTGAGAGATGGCGTTCCAGATGTGTTTTAGAGCTAGAAATAGC |
| OPO42 | FWD | dDNA | *ECE1* dDNA (deletion) | AACAAACAACTTTCCTTTATTTTACTACCAACTATTTTCCATTCGTTAAACTCATGCTCAGCAGATAAAAATTTGTTTTCCACAAGCTTAATCTTTTATT |
| OPO43 | REV | dDNA | *ECE1* dDNA (deletion) | AATAAAAGATTAAGCTTGTGGAAAACAAATTTTTATCTGCTGAGCATGAGTTTAACGAATGGAAAATAGTTGGTAGTAAAATAAAGGAAAGTTGTTTGTT |
| OPO48 | FWD | cPCR/Sequencing | *ECE1* flanking primer | CCCAATAGGATCAGTAAATTCTGC |
| OPO49 | REV | cPCR | *ECE1* flanking primer | TTTACACGCAAAGAGACACC |
| OPO52 | FWD | cPCR | *ECE1* ORF specific primer | TGTTGCTTCTACCAAGAGAGATG |
| OPO53 | REV | cPCR | *ECE1* ORF specific primer | CAATACCGACAGTTTCAATGCTC |
| OPO33 | FWD | gRNA | *HGC1* ORF gRNA | CGTAAACTATTTTTAATTTGGATTTGGAGCCAGAAGCTTGGTTTTAGAGCTAGAAATAGC |
| OPO34 | FWD | dDNA | *HGC1* dDNA (deletion) | AATATCTAGGGTTTCCATTCACATATACACATATAAACATATATTAATAGGAGAAAGAGAGAAAGATAAAGTAGAGAATGGAGAATGGAGAAAGATGTTG |
| OPO35 | REV | dDNA | *HGC1* dDNA (deletion) | CAACATCTTTCTCCATTCTCCATTCTCTACTTTATCTTTCTCTCTTTCTCCTATTAATATATGTTTATATGTGTATATGTGAATGGAAACCCTAGATATT |
| OPO36 | FWD | gRNA | *HGC1* gRNA (mADD-TAG) | CGTAAACTATTTTTAATTTGACATATAAACATATATTAATGTTTTAGAGCTAGAAATAGC |
| OPO65 | FWD | cPCR/Sequencing | *HGC1* Flanking specific primers | GCATCAAACCAATACCCAACAC |
| OPO66 | REV | cPCR | *HGC1* Flanking specific primers | AGGCAAAACGATAAGCAACAC |
| OPO106 | FWD | cPCR/Sequencing | *HGC1* FWD Flanking | CTTCCATACAAGAAGAGTCC |
| OPO107 | REV | cPCR/Sequencing | *HGC1* REV Flanking | GGATACTTTCCAGTAGTGTA |
| OPO108 | FWD | cPCR | *HGC1* FWD ORF | CAGACCATCACCACCAATGA |
| OPO109 | REV | cPCR | *HGC1* REV ORF | CTGGAGTAGTAGAGCCAGTAGAA |
| OPO80 | REV | gRNA | *SAP2* (5' end) | CGTAAACTATTTTTAATTTGGTTCATTGTGTAAAGTCACTGTTTTAGAGCTAGAAATAGC |
| OPO91 | FWD | dDNA | *SAP2* dDNA (adding stop codon and Sap*I* site + GG) using gRNA OPO80) | TTACTAATGGTCAAGAAGGTAAAACTTCCAAAAGACAAGCTGTCTAAGCTCTTCGGAAGTCACTTATGCTGCTGATATTACCGTTGGATCCAATAATCAA |
| OPO92 | REV | dDNA | *SAP2* dDNA (adding stop codon and SAPI site + GG) using gRNA OPO80) | TTGATTATTGGATCCAACGGTAATATCAGCAGCATAAGTGACTTCCGAAGAGCTTAGACAGCTTGTCTTTTGGAAGTTTTACCTTCTTGACCATTAGTAA |
| OPO93 | FWD | gRNA | *SAP2* gRNA ADD-BACK | CGTAAACTATTTTTAATTTGGACAAGCTGTCTAAGCTCTTGTTTTAGAGCTAGAAATAGC |
| OPO31 | FWD | cPCR | *SAP2* forward ORF specific primer | TTATTAGTCGATGCTACTCCAAC |
| OPO32 | REV | cPCR | *SAP2* reverse ORF specific primer | AATTCGGAAGCTGGAACG​ |
| OPO62 | FWD | cPCR/Sequencing | *SAP2* Flanking Primers | TGCATTTGAATAAACGGCATC |
| OPO90 | REV | cPCR | *SAP2* Flanking Primers | TGACAGTTTTATTTGATGCTTGTG |
| ACT1 | FWD | qPCR | Actin primer qPCR | ACTACCATGTTCCCAGGTATTG |
| ACT1 | REV | qPCR | Actin primer qPCR | CCACCAATCCAGACAGAGTATT |
| OPO73 | FWD | qPCR | *ALS3* Primer 1 | CTCCAGGTGAAACCGATACTG |
| OPO74 | REV | qPCR | *ALS3* Primer 2 | ACAGTGGTGGTTGTAGTGTAAG |
| *ECE1*_fw | FWD | qPCR | *ECE1* | CTTTATCTTCTCAAGCTGC |
| *ECE1*_rev | REV | qPCR | *ECE1* | CAACAACAGAATCAATATCTTC |
| OPO71 | FWD | qPCR | *HGC1* Primer 1 | TGGCGGGTTTGGTAATAGTG |
| OPO72 | REV | qPCR | *HGC1* Primer 2 | ACTGGAGTAGTAGAGCCAGTAG |
| OPO119 | FWD | qPCR | *SAP1* | TTTCATCGCTCTTGCTATTGCTT |
| OPO120 | REV | qPCR | *SAP1* | TGACATCAAAGTCTAAAGTGACAAAACC |
| OPO123 | FWD | qPCR | *SAP3* | GGACCAGTAACATTTTTATGAGTTTTGAT |
| OPO124 | REV | qPCR | *SAP3* | TGCTACTCCAACAACTTTCAACAAT |
| OPO125 | FWD | qPCR | *SAP4* | CAATTTAACTGCAACAGGTCCTCTT |
| OPO126 | REV | qPCR | *SAP4* | AGATATTGAGCCCACAGAAATTCC |
| OPO127 | FWD | qPCR | *SAP5* | CATTGTGCAAAGTAACTGCAACAG |
| OPO128 | REV | qPCR | *SAP5* | CAGAATTTCCCGTCGATGAGA |
| OPO129 | FWD | qPCR | *SAP6* | CCTTTATGAGCACTAGTAGACCAAACG |
| OPO130 | REV | qPCR | *SAP6* | TTACGCAAAAGGTAACTTGTATCAAGA |
| OPO131 | FWD | qPCR | *SAP7* | ATGGACACAGTGTGAAATATGAAGTG |
| OPO132 | REV | qPCR | *SAP7* | TCAGTGGAGGATGGACCATTAGA |
| OPO133 | FWD | qPCR | *SAP8* | TCTCAAGAAATTATCCCCCAAAATA |
| OPO134 | REV | qPCR | *SAP8* | TCGGTTCCATTATCAGAATTTGTTC |
| OPO135 | FWD | qPCR | *SAP9* | ATTTACTCCACAGTTTATATCACTGAAGGT |
| OPO136 | REV | qPCR | *SAP9* | CCACCAGAACCACCCTCAGTT |
| OPO137 | FWD | qPCR | *SAP10* | CCCGGTATCCAATAGAATCGAA |
| OPO138 | REV | qPCR | *SAP10* | TCAGTGAATGTGACGAATTTGAAGA |
| OPO150 | FWD | qPCR | *ALS1* | GACTAGTGAACCAACAAATACCAGA |
| OPO151 | REV | qPCR | *ALS1* | CCAGAAGAAACAGCAGGTGA |
| OPO152 | FWD | qPCR | *ALS2* | CCAAGTATTAACAAAGTTTCAATCACTTAT |
| OPO153 | REV | qPCR | *ALS2* | TCTCAATCTTAAATTGAACGGCTTAC |
| OPO154 | FWD | qPCR | *ALS3* | CCACTTCACAATCCCCATC |
| OPO155 | REV | qPCR | *ALS3* | CAGCAGTAGTAGTAACAGTAGTAGTTTCATC |
| OPO156 | FWD | qPCR | *ALS4* | CCCAGTCTTTCACAAGCAGTAAAT |
| OPO157 | REV | qPCR | *ALS4* | GTAAATGAGTCATCAACAGAAGCC |
| OPO158 | FWD | qPCR | *ALS5* | TGACTACTTCCAGATTTATGCCGAG |
| OPO159 | REV | qPCR | *ALS5* | ATTGATACTGGTTATTATCTGAGGGAGAAA |
| OPO160 | FWD | qPCR | *ALS6* | GACTCCACAATCATCTAGTAGCTTGGTTT |
| OPO161 | REV | qPCR | *ALS6* | CAATTGTCACATCATCTTTTGTTGC |
| OPO162 | FWD | qPCR | *ALS7* | GAAGAGAACTAGCGTTTGGTCTAGTTGT |
| OPO163 | REV | qPCR | *ALS7* | TGGCATACTCCAATCATTTATTTCA |
| OPO164 | FWD | qPCR | *ALS9* | CCATATTCAGAAACAAAGGGTTC |
| OPO165 | REV | qPCR | *ALS9* | AACTGAAACTGCTGGATTTGG |
| *CXCL1* | FWD | Murine qPCR Primer | *CXCL1* | AAAGATGCTAAAAGGTGTC |
| *CXCL1* | REV | Murine qPCR Primer | *CXCL1* | GTATAGTGTTGTCAGAAGCC |
| *IL-6* | FWD | Murine qPCR Primer | *IL-6* | AAGAAATGATGGATGCTACC |
| *IL-6* | REV | Murine qPCR Primer | *IL-6* | GAGTTTCTGTATCTCTCTGAAG |
| *IL17A* | FWD | Murine qPCR Primer | *IL17A* | CCCCTTTACACCTTCTTTTC |
| *IL17A* | REV | Murine qPCR Primer | *IL17A* | ACGTTTCTCAGCAAACTTAC |
| *TNF* | FWD | Murine qPCR Primer | *TNF* | CTATGTCTCAGCCTCTTCTC |
| *TNF* | REV | Murine qPCR Primer | *TNF* | CATTTGGGAACTTCTCATCC |
| *S100A9* | FWD | Murine qPCR Primer | *S100A9* | AGCCTTGAAGAGCAAGAAG |
| *S100A9* | REV | Murine qPCR Primer | *S100A9* | GTCAGGGTGTCCTTCCTTCC |
| *GAPDH* | FWD | Murine qPCR Primer | *GAPDH* | CTAATGACCACAGTCCATTC |
| *GAPDH* | REV | Murine qPCR Primer | *GAPDH* | GATGGGATGATGTTTTGGTG |

**Table S4. Reagent concentrations & thermocycler conditions for gRNA stitching.**

| **Fragment A** | |
| --- | --- |
| ***Reagents*** | ***PCR Conditions*** |
| 75.5 μL H_2_O | 1) 98°C for 30 s |
| 20 μL 5 x Phusion HF Buffer | 2) 98°C for 20 s |
| 2 μL dNTP mix (10 mM each) | 3) 58°C for 20 s |
| 1 μL pADH110 (1 ng/μL) | 4) 72°C for 30 s |
| 0.5 μL AHO1096 (100 μM) | Return to Step 2 for 30 cycles |
| 0.5 μL AHO1098 (100 μM) | 5) End |
| 0.5 μL Phusion Polymerase |  |
| **Fragment B** | |
| ***Reagents*** | ***PCR Conditions*** |
| 13.3 μL H_2_O | 1) 98°C for 30 s |
| 4 μL 5 x Phusion HF Buffer | 2) 98°C for 20 s |
| 0.4 μL dNTP mix (10 mM each) | 3) 65°C for 20 s |
| 0.2 μL pADH119 (1 ng/μL) | 4) 72°C for 30 s |
| 1 μL AHO1097 (100 μM) | Return to Step 2 for 10 cycles, reducing annealing temperature by 1°C/cycle |
| 1 μL Custom gRNA (10 μM) | 5) 98°C for 20 s |
| 0.1 μL Phusion Polymerase | 6) 55°C for 20 s |
|  | 7) 72°C for 30 s |
|  | Return to Step 5 for 25 cycles |
|  | 8) End |
| **Fragment C Part 1** | |
| ***Reagents*** | ***PCR Conditions*** |
| 74.5 μL H_2_O | 1) 98°C for 30 s |
| 20 μL 5 x Phusion HF Buffer | 2) 98°C for 20 s |
| 2 μL dNTP mix (10 mM each) | 3) 58°C for 20 s |
| 1 μL Universal Fragment A | 4) 72°C for 1 min |
| 1 μL Unique Fragment B | Return to Step 2 for 5 cycles |
| 0.5 μL Phusion Polymerase | 5) End & Proceed to **Fragment C Part 2** |
| **Fragment C Part 2** | |
| ***Reagents*** | ***PCR Conditions*** |
| 99 μL Fragment C Part 1 PCR Product | 1) 98°C for 30 s |
| 0.5 μL AHO1237 (100 μM) | 2) 98°C for 20 s |
| 0.5 μL AHO1238 (100 μM) | 3) 66°C for 20 s |
|  | 4) 72°C for 1 min |
|  | Return to Step 2 for 30 cycles |
|  | 6) End |

**Table S5. Reagent concentrations & thermocycler conditions for cPCR.**

| ***Reagents (for 15 μL reaction)*** | ***PCR Conditions*** |
| --- | --- |
| 11.7 μL H_2_O | 1) 95°C for 1 min |
| 1.5 DreamTaq Green 10 x Buffer | 2) 95°C for 30 s |
| 0.15 μL dNTP mix (10 mM each) | 3) Primer pair specific annealing temperature for 30 s |
| 0.75 μL Forward Oligo (10 μM) | 4) 72°C for 1 min |
| 0.75 μL Reverse Oligo (10 μM) | Return to Step 2 for 30 cycles |
| 0.15 μL DreamTaq Polymerase | 5) 72°C for 5 min |
| Pipette tip dab of fungal colony | 6) End |

**Table S6. Reagent concentrations & thermocycler conditions for gDNA PCR.**

| ***Reagents (for 50 μL reaction)*** | ***PCR Conditions*** |
| --- | --- |
| 32.5 μL H_2_O | 1) 98°C for 30 s |
| 10 μL 5 x Phusion HF Buffer | 2) 98°C for 10 s |
| 1 μL dNTP mix (10 mM each) | 3) Primer pair specific annealing temperature for 30 s |
| 2.5 μL Forward Primer (10 μM) | 4) 72°C for 15-30 s/kb |
| 2.5 μL Reverse Primer (10 μM) | Return to Step 2 for 30 cycles |
| 1 μL gDNA (50 ng/μL) | 5) 72°C for 5 min |
| 0.5 μL Phusion Polymerase | 6) End |
